# Genome-level diversification of eight ancient tea populations in the Guizhou and Yunnan regions identifies candidate genes for core agronomic traits

**DOI:** 10.1038/s41438-021-00617-9

**Published:** 2021-08-10

**Authors:** Litang Lu, Hufang Chen, Xiaojing Wang, Yichen Zhao, Xinzhuan Yao, Biao Xiong, Yanli Deng, Degang Zhao

**Affiliations:** 1 College of Tea Science, Guizhou University, Guiyang, 550025 People’s Republic of China; 2 College of Life Sciences and The Key Laboratory of Plant Resources Conservation and Germplasm Innovation in the Mountainous Region (Ministry of Education), Institute of Agro-Bioengineering, Guizhou University, Guiyang, 550025 People’s Republic of China; 3 Guizhou Academy of Agricultural Sciences, Guiyang, 550025 People’s Republic of China

**Keywords:** Genome, Plant genetics

## Abstract

The ancient tea plant, as a precious natural resource and source of tea plant genetic diversity, is of great value for studying the evolutionary mechanism, diversification, and domestication of plants. The overall genetic diversity among ancient tea plants and the genetic changes that occurred during natural selection remain poorly understood. Here, we report the genome resequencing of eight different groups consisting of 120 ancient tea plants: six groups from Guizhou Province and two groups from Yunnan Province. Based on the 8,082,370 identified high-quality SNPs, we constructed phylogenetic relationships, assessed population structure, and performed genome-wide association studies (GWAS). Our phylogenetic analysis showed that the 120 ancient tea plants were mainly clustered into three groups and five single branches, which is consistent with the results of principal component analysis (PCA). Ancient tea plants were further divided into seven subpopulations based on genetic structure analysis. Moreover, it was found that the variation in ancient tea plants was not reduced by pressure from the external natural environment or artificial breeding (nonsynonymous/synonymous = 1.05). By integrating GWAS, selection signals, and gene function prediction, four candidate genes were significantly associated with three leaf traits, and two candidate genes were significantly associated with plant type. These candidate genes can be used for further functional characterization and genetic improvement of tea plants.

## Introduction

The leaves of the tea plant *Camellia sinensis* (L.) O. Kuntze var. *sinensis* (2*n* = 2*x* = 30) are used to produce different kinds of tea, making tea an important economic crop worldwide. With its attractive aroma and pleasant taste^1,2^, tea is the most popular nonalcoholic caffeine-containing beverage in the world and is consumed daily by more than three billion people across 160 countries. Tea beverages are rich in beneficial compounds, such as polyphenols, caffeine, theanine, vitamins, polysaccharides, volatile oils, and minerals, which have been shown to reduce the risk of developing cancer and cardiovascular, cerebrovascular, and nervous system diseases^3–7^. The Camellia species encompasses highly diverse crops that produce secondary metabolites in the buds and young leaves, which were targets of selection during the process of domestication. Thus, the leaf inclusions and morphological characteristics of tea plants can be used as an indicator of the selection process in tea plant breeding. At present, cultivated tea plants include two main varieties: *C. sinensis* var. *sinensis* (Chinese type tea; CSS) and *C. sinensis* var. *assamica* (Assam type tea; CSA)^8,9^.

Analyses of genome-wide genetic diversity and the identification of genes associated with excellent traits that contribute to domestication and improvement play an essential role in the breeding of superior varieties^10–12^. Genome-wide association studies (GWAS) using whole-genome resequencing identified new genes influencing agronomic traits in crop plants^13–17^. The release of the tea genome database laid the foundation for genome resequencing and GWAS^18^. Resequencing dozens of tea cultivars has allowed preliminary understanding of the genetic variation patterns during tea plant domestication and varietal improvement; however, the high degree of heterozygosity in tea cultivars has hindered the correlation of selected loci with improvement and domestication related traits.

Ancient tea plants grew naturally for hundreds of years without any human cultivation, and the genetic diversity of these plants is important for studying the origin, spread, and classification of tea plants. The ancient tea plants were mainly distributed in the Yunnan-Guizhou Plateau. In this study, we resequenced (more than 10x) a large set of plants representative of the various morphotypes of ancient tea plants from the Yunnan-Guizhou Plateau. Phylogenetic relationships and population structure were assessed and GWAS was performed. RT-qPCR was used to verify the expression pattern of genes mined using GWAS in representative ancient tea plants from eight ancient tea populations. Our findings provide useful information for future breeding and molecular identification of tea plants.

## Results

### Sequencing and variant discovery

**Fig. 1:**
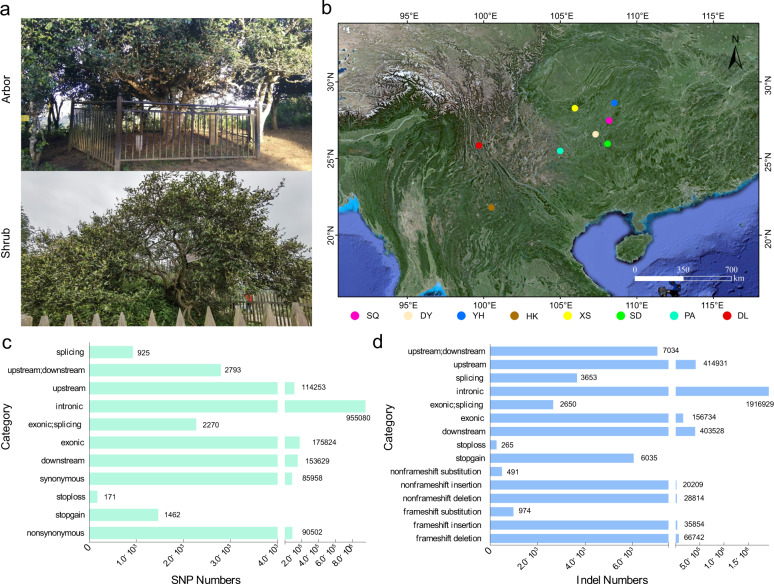
Geographic distribution of 120 ancient tea plants and statistical analysis of SNPs and indels in the tea genome. **a** Plant type of ancient tea plants. **b** The geographic distribution of the 120 ancient tea plants, each of which is represented by a dot on the map of China. Xishui (XS), Pu’an (PA), Yanhe (YH), Shiqian (SQ), Duyun (DY), Sandu (SD), Dali (DL), and Menghai (HK). **c** Statistical analysis of SNPs in the tea genome. **d** Statistical analysis of indels in the tea genome

A total of 120 ancient tea plants from eight groups, including six groups containing 90 individuals from Guizhou Province and two groups containing 30 individuals from Yunnan Province, China, were evaluated in the present study. The geographic distributions of these plants are Xishui (XS), Pu’an (PA), Yanhe (YH), Shiqian (SQ), Duyun (DY), and Sandu (SD) in Guizhou and Lincang (DL) and Menghai (HK) in Yunnan. Detailed information on the agronomic characteristics of the 120 ancient tea plants was obtained based on Chen’s study (Fig. 1a, b and Tables S1 and S2)^19^.

Resequencing of the 120 ancient tea plants using the Illumina HiSeq 2000 sequencing platform produced over 5.013 billion raw 150-bp paired-end reads, resulting in 5.01 Tb of clean data with an average coverage depth of more than 10x (Table S3). Our resequenced reads were mapped onto the published *C. sinensis* var. *sinensis* genome^18,20,21^. A total of 411,990,204 single nucleotide polymorphisms (SNPs, Fig. 1c) and 18,880,978 indels (insertions and deletions, range 1−54 bp, mean 6.6 bp, Fig. 1d) were identified. Of the 8,082,370 filtered SNPs (coverage depth ≥10, MAF <0.05, and miss rate ≤0.1), 1,404,774 and 175,824 SNPs were distributed in noncoding and coding sequences, respectively. Moreover, 90,502 nonsynonymous SNPs (nsSNPs) were identified in 19,793 genes, and 10,596 frameshift indels were identified in 26,943 genes (Fig. 1c, d). The 6300 variants had a large effect, including SNPs causing premature stop codons or longer-than-usual transcripts and indels resulting in frameshifts, the introduction of stop codons, or other disruptions to protein-coding sequences. To evaluate the selective constraints on ancient tea plants in their natural habitat, the ratio of nonsynonymous to synonymous SNPs (dN/dS) was calculated and found to be 1.05. In addition, among all identified SNPs, 2.1% were located in coding regions: 1.12% were nonsynonymous and 0.98% were synonymous (Table S4). This result showed that the proportion of nsSNPs in the coding regions of ancient tea plants was significantly lower than that detected in pear (7.7%), apple (10.5%), and soybean (1.9%), suggesting that less genetic variation occurs in the coding regions of ancient tea plants than in that of fruit trees and some annual crops^22–24^. Moreover, the accuracy of SNP genotyping in randomly selected genomic regions containing a single SNP site was assessed by PCR and Sanger sequencing, revealing an SNP genotype accuracy as high as 98.1%.

### Phylogenetic analysis and population structure of ancient tea plants

**Fig. 2:**
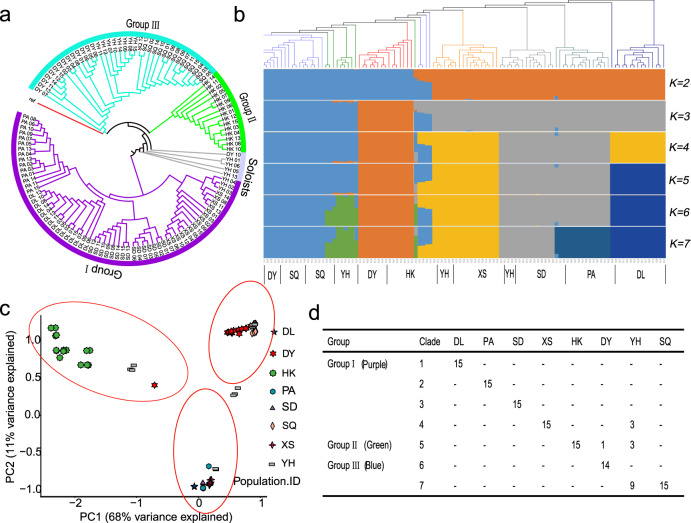
Population genetic structure of 120 ancient tea plant samples. **a** NJ tree of 120 ancient tea plants inferred from SNPs at fourfold degenerate sites. A phylogenetic tree was constructed using MEGA software with default parameters. **b** Model-based Bayesian clustering of 120 ancient tea plants performed using ADMIXTURE Version 1.3.0 with the number of ancestry kinships (K) set to 2–7. Each accession is denoted by a vertical bar composed of different colors corresponding to its proportion of genetic ancestry from each of these populations. **c** Principal component analysis of the 120 ancient tea plants. **d** The geographic origin of each accession in the seven clades

To explore the phylogenetic relationships among the 120 ancient tea plants, a phylogenetic tree was constructed by the neighbor-joining (NJ) method using 8,082,370 SNPs. According to the phylogenetic relationships, the 120 ancient tea plants were mainly clustered into three groups (I−III) and five single branches (Fig. 2a). Among them, group I contained all the members of XS, DL, SD, and PA and three members of YH; group II contained all the members of HK; and group III contained members of DY, SQ, and YH and was located close to the cultivated tea plant in the phylogenetic tree (Table S5). This finding is consistent with those of the previous studies^25^. Changes in population structure were further assessed under different *K* values (Fig. 2b). Analysis of cross-validation error (CV error) revealed that seven populations (*K* = 7) represented the best model for these 120 individual ancient tea plants, while the value of CV error changed little as *K* increased from 2 to 7. At *K* = 2, the HK members were separated from the main groups. The population structure at *K* = 3 was consistent with the three clustered groups in the phylogenetic tree. The XS and DL members were clustered together away from the main groups at *K* = 4, and at *K* = 5 and 6, the DL and some of the YH members were clearly separated from the main groups. These results are not only consistent with the geographic distribution of ancient tea plants but are supported by the phylogenetic analysis, indicating that the species in different subgroups (DY, SQ, YH, HK, XS, SD, PA, and DL) from relatively close areas had common geographic origins and that species from the different geographic areas developed independently. The changes in the population structure among the 120 ancient tea plants were mainly related to members of XS, SD, PA, and DL. To identify potential population stratification, principal component analysis (PCA) was used to explore relationships among the 120 ancient tea plants using ~8 M SNPs (Fig. 2c and Fig. S1). PCA revealed three major clusters corresponding to clusters 1−3 from the phylogenetic tree, which further verifies the accuracy of the phylogenetic tree grouping (Fig. 2a, d). For instance, the PA, SD, XS, and DL samples were clustered together to form cluster 1, the HK samples were clustered together to form cluster 2, and the YH, SQ, and DY samples were clustered together to form cluster 3.

### Population divergence among ancient tea plants

**Fig. 3:**
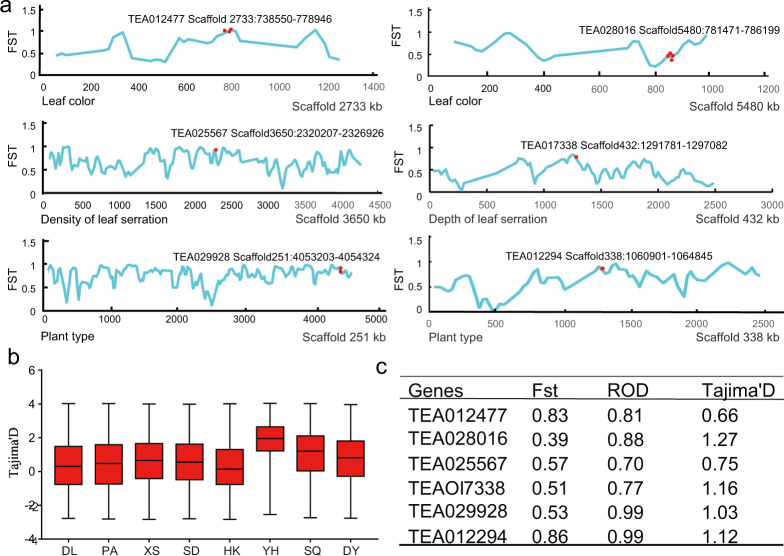
Population sweeps during local adaptation among ancient tea plants from the different areas. **a** The distribution of FST along the scaffold. **b** The distribution of Tajima’s D along the scaffold. **c** Selection signals estimated by the three measures for seven candidate genes for leaf and plant type

Our phylogenetic analysis revealed that genetically close relatives may have similar geographic origins (Fig. 2a, b). Moreover, ancient tea plants from the same place showed similar agronomic traits. For example, most ancient tea plants in DY and SQ were shrub-type plants, whereas those in SD, DL, PA, XS, HK, and YH were tree-type plants. Most ancient tea plants in SQ had dark green leaves, while members of the other groups had light green leaves, and some ancient tea trees in PA had purple leaves. Therefore, some traits and their controlling genes underwent natural screening during the process of geographic isolation. The pairwise population divergence (fixation index: FST) across the PA, DL, DY, and SQ subgroups was analyzed (Fig. 3a, c). The mean FST among the ancient tea plants in DL and DY was 0.745, suggesting obvious population divergence, which was followed by a mean FST of 0.217 among the plants in DL and HK. However, the mean FST among the ancient tea plants in DY and SQ was 0.08, suggesting little population divergence.

Tajima’s D was used to evaluate whether the observed nucleotide diversities showed evidence of deviation from neutrality. Some regions were significantly different from zero, indicating natural or artificial selection (Fig. 3b). Of these, the D values from the SD, DL, PA, DY, XS, HK, YH, and SQ genomes were mostly positive, indicating a predominance of intermediate-frequency SNPs in these subgenomes. The genome-wide nucleotide diversity (ϴπ) across all ancient tea plants was 6.1 × 10^−3^. This value was higher than that of other perennial crops, such as peach (1.5 × 10^−3^), cassava (2.6 × 10^−3^), and pear (5.5 × 10^−3^), but lower than that reported for date palm (9.2 × 10^−3^)^26–28^. The genetic diversity decreased from 6.1 × 10^−3^ in ancient tea plants to 5.15 × 10^−5^ in improved tea cultivars^29^, suggesting the loss of significant genetic diversity during domestication.

### Genome-wide association studies

**Fig. 4:**
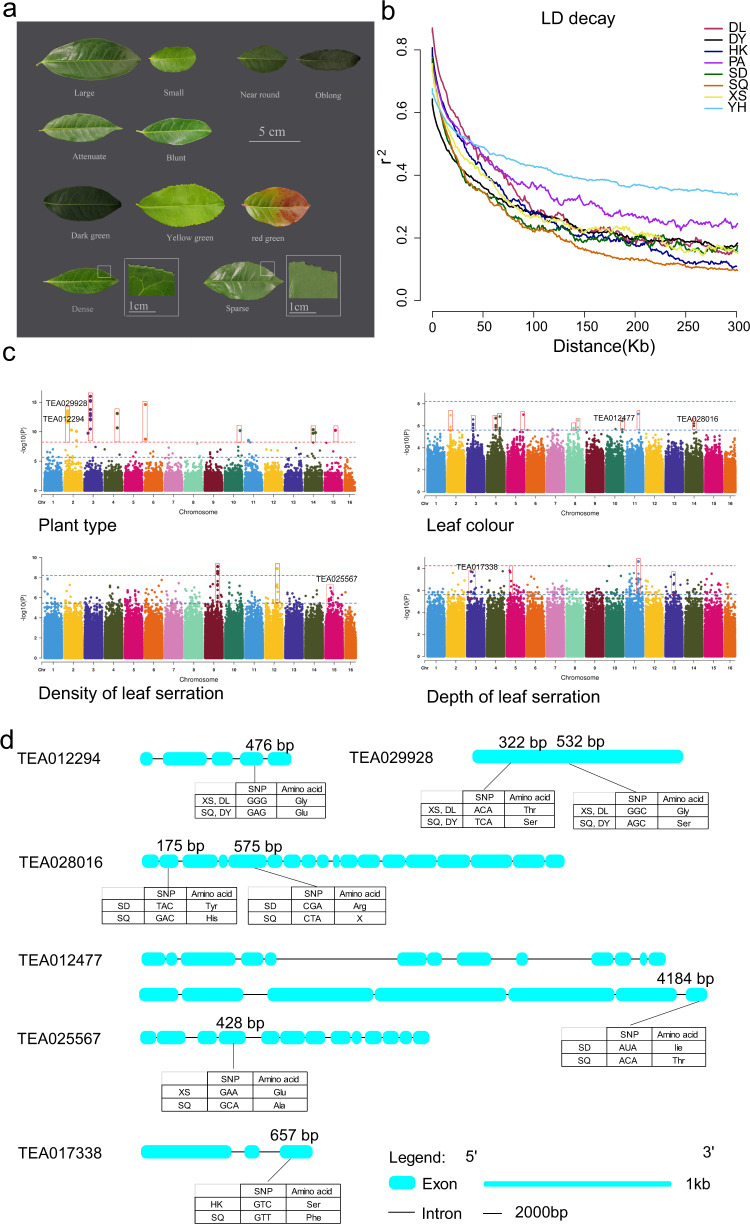
Regions related to leaf traits in the ancient tea plants. **a** Leaf traits of the 120 ancient tea plants. **b** LD analysis of the 120 ancient tea plants. **c** Manhattan plots for four traits. The significance threshold of the –log_10_ *P* value was set at 5.5 (blue). **d** Structure of genes related to leaf traits according to GWAS

Linkage disequilibrium (LD indicated by *r*^*2*^) analysis indicated that the ancient tea plant genome has a relatively short *r*^*2*^ distance and rapid *r*^*2*^ decay (Fig. 4b). The *r*^*2*^ decreased to half its maximum value, at 19.3 kb, which is higher than the *r*^*2*^ in cultivated tea plants (5 kb) but lower than that in ancient tea plants (~40 kb) reported by Xia^9^. Moreover, the ancient tea plants from DL showed the highest *r*^*2*^ value (*r*^*2*^ = 40.0 kb), and those from DY showed the lowest *r*^*2*^ value (*r*^*2*^ = 11.6 kb) (Fig. S2). The LD decay distance for the ancient tea plants was much longer than that for pear (211 bp) and apple (161 bp) but much shorter than that for soybean (150 kb) and rice (123 kb).

Plant domestication conducted over several millennia has resulted in the modification of specific plant traits, including leaf size, shape, texture, width, color, the number of leaf veins, and the density and depth of leaf serration^19,30^. To further screen the candidate genes associated with eleven leaf traits, compression multilocus random mixed linear model analysis was conducted for GWAS using GAPIT software (Fig. 4a). To obtain high-quality SNPs, imputation was performed for the ancient tea plant SNP set, retaining 8,082,370 SNPs with a MAF of 5%. In total, 1176 SNPs were associated with 11 target leaf traits, and 292 loci were involved in regulating plant type. Most of the loci that were associated with ancient leaf traits and plant type are shown here for the first time (Figs. S3−S7 and Table S6).

### Candidate genes involved in the regulation of leaf traits

In tea plants, the leaves are rich in characteristic compounds, such as polyphenols, caffeine, theanine, vitamins, polysaccharides, volatile oils, and minerals. Leaf inclusions are often related to many leaf traits, including leaf size, color, and the number of leaf veins. GWAS signals associated with leaf traits were detected in the present study (Fig. 4c, d and Table 1). Three nsSNPs were identified to have associations with leaf color: two nsSNPs in TEA012477 and one nsSNP in TEA028016 (−log_10_*P* ≥ 8.2). Functional annotation inferred that these two genes encode calmodulin-binding transcription activator 2 and the Cop1/SPA ubiquitin ligase complex, respectively. The latter is involved in the repression of anthocyanin accumulation under low- and high-light conditions in *Arabidopsis* (Table 1). An nsSNP was identified to be significantly (−log_10_*P* ≥ 8.2) related to the density of leaf serration and caused a change from A to C at base 428 in the CDS of TEA025567, resulting in a change from Glu to Ala at residue 143. Moreover, an nsSNP was found to be significantly (−log_10_*P* ≥ 8.2) related to the depth of leaf serration and caused a change from C to T at base 320 in the CDS of TEA017338, resulting in a change from Ser to Phe at residue 107. In addition, the ancient tea plants were classified into shrub and arbor plants. Two nsSNPs were also identified to be significantly associated with plant type and caused a change from A to T at base 322 and from G to A at base 532 in the CDS of TEA029928, resulting in changes from Thr to Ser at residue 108 and from Gly to Ser at residue 178, respectively. Moreover, an nsSNP (G/A) was found at base 476 in the CDS of TEA01294, resulting in a change from Gly to Glu at residue 159. Annotation and functional analysis of homologous genes in *Arabidopsis* showed that these two genes encode an F-box protein and an acyl carrier protein. In *Arabidopsis*, F-box proteins repress ethylene action and promote growth by directing EIN3 degradation (ethylene restricts *Arabidopsis* growth via the epidermis), and acyl carrier proteins control plant architecture by regulating the cytokinin signaling pathway.

**Table 1:** GWAS results for genes associated with different traits

Trait	Gene locus	Exon	SNP sites	Protein	−Log_10_(*P*)	Gene annotation
Leaf color	TEA012477	15	CGT (1826)–GAT	Arg (609)–His	1.16718	Calmodulin-binding transcription activator 2
		18	TCC (3406)–GCC	Ser (1136)–Ala	0.93987	
		20	ATA (4184)–ACA	Ile (1394)–Thr	8.43856	
	TEA028016	2	TAC (175)–GAC	Tyr (59)–His	6.21486	Cop1 ubiquitin ligase complex
		5	CGA (574)-CTA	Arg (192)–Leu	6.21486	
Density of leaf serration	TEA025567	4	GAA (428)–GCA	Glu (143)–Ala	8.90128	Tetratricopeptide repeat (TPR) protein
Depth of leaf serration	TEA017338	3	GTC (657)–GTT	Ser (107)–Phe	7.32429	AT-rich interactive domain protein
Plant type	TEA029928	1	GGC (532)–AGT	Gly (178)–Ser	8.08721	F-box protein
	TEA012294	1	CTG (475)–TGT	Gly (159)–Glu	8.46766	Acyl carrier protein 2

To further verify the differences among the different populations, we selected two genes (TEA012477 and TEA029928) related to leaf color and plant type to detect the distribution of nsSNPs in representative plants of eight different populations. As shown in Fig. S8, we isolated and aligned the homologous sequences of the TEA029928 and TEA012477 genes from eight representative individuals from eight populations. One nsSNP was identified to be significantly (−log_10_*P* ≥ 8.2) related to plant type, causing a change from a C in arbor-type plants (DL, HK, XS, PA, SD, and YH) to an A in shrub-type plants (SQ, DY, and YH) at base 532 in the CDS of TEA029928, resulting in a change from Glu to Ser at residue 178. Moreover, three nsSNPs were significantly (−log_10_*P* ≥ 8.2) related to leaf color, causing changes from G, G, and C in light green plants (DL, HK, XS, PA, SD, YH, and DY) to A, T, and T in dark green plants (SQ) at bases 1826, 3406, and 4184 in the CDS of TEA012477, resulting in a change from Arg, Ala, and Thr to His, Ser and Ile at residues 609, 1136, and 1394.

To further explore the functional differences among different groups, RT-qPCR was used to investigate the expression level of the four representative genes mined using GWAS among different populations with different traits. Our results showed that the expression level of the gene TEA021477 in ancient tea trees with light green leaves was significantly lower than that in ancient tea trees with dark green leaves. The expression level of the gene (TEA029928) related to plant type in shrub-type ancient tea plants was significantly higher than that in arbor-type ancient tea plants (Fig. S9). In addition, a previous study revealed that the density and depth of leaf serration were quantitative traits controlled by multiple genes. Dynamic expression changes in the genes related to leaf serration of different densities and depths were investigated using RT-qPCR, suggesting that these two traits were controlled by multiple genes^31^. The nsSNPs were used as molecular markers to distinguish the difference between shrub- and arbor-type ancient tea plants, which were also used to determine the difference between light green and dark green leaves (Tables S7, S8 and Fig. S9).

## Discussion

Although *C. sinensis* “Fuding Dabaicha” has been widely planted in the southwestern region of China due to its high yield and economic value, there remain many ancient tea plant resources that have not been exploited^8^. In the present study, we generated a dataset encompassing the considerable genomic variation of ancient tea plants, which provided an opportunity to explore the divergence, population structure, and regulatory mechanisms of related traits in ancient tea plants. A previous study suggested that tea plants have diverse origins and that Chinese cultivated tea plants originated from southwestern China and later spread to western Asia^28^. The results of our phylogenetic analysis show that the 120 ancient tea plants were mainly clustered into three groups and five single branches. Three members of YH were clustered together with the ancient tea plants from XS and distributed in group 1, indicating that gene exchange occurred between them, which could be partially attributed to the consistent introgression among the ancient tea plants during the long cultivation process^9^. Our results further reveal that the ancient tea plants in DL and PA are more ancient than those in the other six populations, and the ancient tea plants in DY and SQ are closely related to the cultivated tea plant based on phylogenetic analysis, which is consistent with the findings of the previous studies^28^. It has been demonstrated that ancient tea plants from DY are closer to modern cultivars^32^. Based on the phylogenetic analysis, the ancient tea plants from DY and SQ were a sister branch to the cultivars, which is consistent with the results of a previous study. The ancient tea plants of XS clustered outside of the clade containing the SD, DL, and PA groups, suggesting that the members of XS are more ancient than the members of the SD, DL, and PA groups. Moreover, some members of YH clustered outside the clade containing the HK, DY, and SQ groups, suggesting that some members of YH are more ancient than the members of the HK, DY, and SQ groups.

The results of LD analysis demonstrated that natural selection pressures acted during the evolution and domestication of ancient tea plants. The ancient tea plants in DL showed the highest LD (*r*^*2*^ = 467 kb), indicating that these plants experienced the greatest selection pressure. The results of kinship (*K*) analysis further revealed that the 120 ancient tea plants were clustered into seven subgroups with monophyletic clades from the same or nearby places, indicating a common geographic origin.

Artificial breeding of cultivated tea plants dramatically reduces genetic diversity^9^. As a precious natural resource, ancient tea plants have higher genetic diversity, which is of great value for studying the evolutionary mechanism and diversification of tea plants. This phenomenon is consistent with results in crops such as rice^33^. As expected, a number of outlier regions were identified, and 19 candidate genes were found to contain 107 SNPs associated with plant type and leaf traits, including leaf length, width, size, shape, texture, color, the number of leaf veins, and the density and depth of leaf serration. Among these genes, six genes with eight SNPs were significantly associated with four traits based on KEGG annotation and functional analysis of orthologous genes in *Arabidopsis*, suggesting that these candidate genes screened by GWAS may be involved in regulating the development of leaf-related traits and plant type (Table 1). In *Arabidopsis*, the Cop1/SPA ubiquitin ligase complex is involved in repressing anthocyanin accumulation under low- and high-light conditions and the F-Box protein (corresponding to the functional gene of TEA029928) regulates leaf size^34,35^. Moreover, the soybean stearoyl-acyl carrier protein (corresponding to the functional gene of TEA023604) regulates the different morphological phenotypes of the leaves^36,37^. Furthermore, NADH dehydrogenase (ubiquinone) 1 alpha (corresponding to the functional gene of TEA012294) regulates the different morphologies of the plant (Table 1)^38,39^.

Our study provides a valuable resource for understanding the phylogenetic relationships, population structure, and genetic diversity of ancient tea plants in southwestern China. In addition, candidate genes significantly associated with four important agronomic traits were identified. The significant SNPs associated with favorable variants, selection signals, and candidate genes are a valuable resource for the further improvement of leaf traits and plant type in ancient tea plants.

## Materials and methods

### Plant material and agronomic evaluation

A total of 120 ancient tea plants were selected to represent a broad distribution of geography, morphology, and genetic diversity. These plant materials included 90 individual ancient tea plants from Guizhou, China, and 30 individual ancient tea plants from Yunnan, China, which were classified and standardized according to the book of Chinese tea plants^19^. The different values represent the different qualitative and quantitative traits based on the classification and standardization of tea plants.

### Sequencing, mapping, and SNP calling

Total DNA was extracted using the modified cetyltrimethylammonium bromide (CTAB) method^40^. At least 6 μg of genomic DNA from each individual was used to construct a sequencing library following the manufacturer’s instructions (Illumina Inc.). Paired-end sequencing libraries with an insert size of ~400 bp were sequenced on an Illumina HiSeq 2000 sequencer at BGI company. The draft genome sequence of the tea plant (*C. sinensis* var. *sinensis* cv. shuchazao), downloaded from the TPIA database (http://tpia.teaplant.org/), was used as a reference genome^9,41^. Paired-end reads were mapped to the tea reference genome with BWA (v 0.6.1) software using the default parameters^42^. SAMtools software was used to convert mapping results into the BAM format and to filter the unmapped and nonunique reads^43^. The Picard package was used to filter the duplicated reads^42^. The CoverageBed program in BEDtools v2.17.0 was used to calculate the coverage of sequence alignments^44^. After alignment, SNP calling was conducted per individual using SAMtools^45^. The genotype likelihoods were evaluated from the reads of each individual at each genomic location. A Bayesian approach was used to determine the allele frequencies. SNPs were identified by the samtools mpileup command. To remove false positives, only 8,082,370 high-quality filtered SNPs (coverage depth ≥ 10, MAF < 0.05, and miss rate ≤ 0.1) were used in the subsequent analysis.

### Functional annotation of genetic variants

SNP annotation was conducted based on the draft genome of *C. sinensis* var. *sinensis* using the ANNOVAR package^9,46,47^. According to the annotation information, SNPs were distributed in exonic regions, splicing sites, 5′ UTRs, 3′ UTRs, intronic regions, upstream and downstream regions (which were distributed in 1 kb regions away from the transcription start site), and intergenic regions. Moreover, SNPs in exonic regions were further divided into synonymous SNPs (sSNPs) or nsSNPs. Indels in the coding regions were identified based on frame-shift mutations (3 bp insertion or deletion).

### Phylogenetic tree and population genetics analysis

To explore the phylogenetic relationship of ancient tea plants at the genome-wide level, an NJ tree was constructed using the p-distance in MEGA v7.0 software with bootstrap values determined from 1000 replicates^48^.

Admixture software was used to analyze the population structure of 120 ancient tea plants with *K* values ranging from 2 to 13^28^. PCA was also used to evaluate the genetic structure of the ancient tea populations using GCTA software^49^.

### Linkage disequilibrium analysis

To compare the patterns of LD among the different ancient tea populations, the squared correlation coefficient (*r*^*2*^) between pairwise SNPs was computed using PopLDDecay 3.26 software with default parameters^50^. The average *r*^*2*^ value was calculated for pairwise markers in a 100-kb window and averaged across the whole genome.

### Genome-wide association study

The R package GENESIS v.2.14.157 was employed to perform GWAS between genotypes and phenotypes for all quantitative and qualitative data^51^. The genetic relationship matrix (GRM) can be used as a generalized linear mixed model of random effects to explain population stratification. In the present study, it was used to test the abovementioned association. GCTA v.1.92.158 was applied to calculate the GRM^52^. The count data adopted a Poisson distribution, and the remaining quantitative data adopted a Gaussian distribution. If necessary, a Box−Cox power transformation was used, and normality was verified using a Shapiro–Wilk test. For qualitative traits, a binomial distribution was assumed, while for multiple qualitative traits, each category level was treated as a virtual binary variable. Quantile–quantile plots were used to evaluate the GWAS model (Figs. S4−S8 a, b). The GEC (Genetic Type 1 Error Calculator) v. 0.259 was used to estimate the significance level of correlation^53^. Compression multilocus random mixed linear model analysis was conducted for GWAS using GAPIT software^54^. The GWAS correction threshold was 8.2 (−log_10_ (0.05/8082370)) (Manhattan plot, red dotted line).

Population fixation statistics (FST) and reduction of diversity (ROD) were calculated for nonoverlapping genomic intervals in 1-kb windows using VCFtools^55^. All the output results of ROD and FST were standardized and transformed into *z*-scores using a 100-kb sliding window with a 10-kb step size. The outlier windows of ROD and FST with high values were used to identify candidate genes based on *z*-tests with a significance level of α = 0.05 corresponding to a *z*-score of 1.645. The population genetics statistic Tajima’s D was calculated directly from short-read alignments using ANGSD with nonoverlapping 10-kb intervals (version 0.609)^56^.

### Extraction of RNA and RT-qPCR analysis

The total RNA of ancient tea plants was isolated using a Huayueyang Plant RNA Extraction Kit (Quick RNA isolation Kit; Haidian District, Beijing). The expression patterns of four genes identified by GWAS were measured using RT-qPCR. RT-qPCR was performed using SYBR Premix Ex Tag (TaKaRa) using cDNA as the template. The results were analyzed using the −ΔΔCT method with GAPDH gene expression as an internal reference. Three biological and three technical replicates were used^57^.

## Supplementary information

Genetic diversity of ancient tea plants by GWAS analysis supplement

## Data Availability

The datasets presented in this study can be found in online repositories. The names of the repositories and accession numbers can be found below: PRJNA716079 (Table S9).
