# Supplementary material for: Genome-level diversification of eight ancient tea populations in the Guizhou and Yunnan regions identifies candidate genes for core agronomic traits
Source: Hortic Res. 2021 Aug 10;8:190. doi: 10.1038/s41438-021-00617-9 (PMC8355299; doi:10.1038/s41438-021-00617-9)
Supplement: Supplementary file 1 — Genetic diversity of ancient tea plants by GWAS analysis supplement [file 41438_2021_617_MOESM1_ESM.docx]

**The genome-level diversification of eight ancient tea populations in the Guizhou and Yunnan regions identifies candidate genes for core agronomic traits**

**Running title (no more than 50 characters):** Genetic diversity of ancient tea plants by GWAS analysis

Litang Lu^1,3^, Hufang Chen^1,3^, Xiaojing Wang^1^, Yichen Zhao^1,3^, Xinzhuan Yao^1^, Biao Xiong^1^, Yanli Deng^1^, Degang Zhao^2,3*^

^1^College of Tea Science, Guizhou University, Guiyang, 550025, People’s Republic of China

^2^Guizhou Academy of Agricultural Sciences, Guiyang, 550025, People’s Republic of China

^3^College of Life Sciences and The Key Laboratory of Plant Resources Conservation and Germplasm Innovation in the Mountainous Region (Ministry of Education), Institute of Agro-Bioengineering, Guizhou University, Guiyang, 550025, People’s Republic of China

Litang Lu, Hufang Chen and Xiaojing Wang contributed equally to this work and should be considered co-first authors

Litang Lu: ltlv@gzu.edu.cn

Hufang Chen: 15761629978@163.com

Xinzhuan Yao: [xzyao@gzu.edu.cn](mailto:xzyao@gzu.edu.cn)

Xiaojing Wang: [173160235@qq.com](mailto:173160235@qq.com)

Biao Xiong: bxiong@gzu.edu.cn

Yanli Deng: yldeng@gzu.edu.cn

*Correspondence:

E-mail: dgzhao@gzu.edu.cn (Degang. Zhao); Telephone number: +8613885012693

**Table S1 Morphological characteristics of tea plants and numerical value**

| Traits | Quantification of qualitative trait | | | | |
| --- | --- | --- | --- | --- | --- |
|  | 1 | 2 | 3 | 4 | 5 |
| Leaf area /cm2 | Small | Medium | Large | Extra large | Super extra large |
| Leaf shape | Near round | Elliptic | Oblong | Lanceolate | - |
| Leaf colour | Yellow green | Light green | Medium green | Dark green | Purple green |
| Leaf apex shape | Attenuate | Obtuse | Blunt | - | - |
| Density of leaf serration | Sparse | Medium | Dense | - | - |
| Depth of leaf serration | Shallow | Medium | Deep |  | - |
| Leaf texture | Soft | Medium | Hard | - | - |
| Plant type | Arbor | Shrub | - | - | - |

**Table S2 The information of 120 individual ancient tea plants**

| Number | Leaf long | Leaf width | Leaf area /cm2 | Leaf size | Leaf Shape Index | Leaf shape | Leaf colour | Leaf texture | Leaf apex shape | Density of leaf serration | Depth of leaf serration | Number of vein pairs | Plant type |
| --- | --- | --- | --- | --- | --- | --- | --- | --- | --- | --- | --- | --- | --- |
| XS_01 | 9.81 | 4.22 | 28.97874 | 2 | 2.324645 | 2 | 3 | 2 | 1 | 2 | 1 | 12 | 1 |
| XS_02 | 5.08 | 4.31 | 15.32636 | 1 | 1.178654 | 1 | 3 | 2 | 1 | 1 | 1 | 10 | 1 |
| XS_03 | 12.54 | 4.93 | 43.27554 | 3 | 2.543611 | 3 | 3 | 2 | 1 | 2 | 1 | 11 | 1 |
| XS_04 | 14.31 | 7.27 | 72.82359 | 4 | 1.968363 | 1 | 3 | 2 | 1 | 2 | 1 | 10 | 1 |
| XS_05 | 10.66 | 3.82 | 28.50484 | 2 | 2.790576 | 3 | 2 | 2 | 1 | 2 | 1 | 10 | 1 |
| XS_06 | 9.57 | 4.74 | 31.75326 | 2 | 2.018987 | 2 | 3 | 2 | 1 | 1 | 1 | 9 | 1 |
| XS_07 | 8.81 | 4.97 | 30.64999 | 2 | 1.772636 | 1 | 3 | 2 | 1 | 1 | 1 | 10 | 1 |
| XS_08 | 13.45 | 7.84 | 73.8136 | 4 | 1.715561 | 1 | 3 | 2 | 1 | 1 | 1 | 8 | 1 |
| XS_09 | 13.67 | 7.55 | 72.24595 | 4 | 1.810596 | 1 | 3 | 2 | 1 | 1 | 1 | 10 | 1 |
| XS_10 | 12.07 | 5.78 | 48.83522 | 3 | 2.088235 | 2 | 3 | 2 | 1 | 1 | 1 | 10 | 1 |
| XS_11 | 12.39 | 5.68 | 49.26264 | 3 | 2.181338 | 2 | 3 | 2 | 1 | 1 | 1 | 9 | 1 |
| XS_12 | 11.07 | 4.71 | 36.49779 | 2 | 2.350318 | 2 | 3 | 2 | 1 | 1 | 1 | 10 | 1 |
| XS_13 | 14.24 | 8.58 | 85.52544 | 4 | 1.659674 | 1 | 3 | 2 | 1 | 1 | 1 | 10 | 1 |
| XS_14 | 9.07 | 4.96 | 31.49104 | 2 | 1.828629 | 1 | 3 | 1 | 1 | 1 | 1 | 11 | 1 |
| XS_15 | 13.15 | 7.72 | 71.0626 | 4 | 1.703368 | 1 | 3 | 3 | 1 | 1 | 2 | 8 | 1 |
| PA_01 | 16.93 | 6.02 | 71.34302 | 4 | 2.812292 | 3 | 3 | 2 | 1 | 1 | 2 | 12 | 1 |
| PA_02 | 16.08 | 6.99 | 78.67944 | 4 | 2.300429 | 2 | 3 | 2 | 1 | 3 | 2 | 13 | 1 |
| PA_03 | 13.18 | 5.14 | 47.42164 | 3 | 2.564202 | 3 | 2 | 3 | 1 | 3 | 2 | 13 | 1 |
| PA_04 | 14.66 | 5.7 | 58.4934 | 3 | 2.57193 | 3 | 3 | 2 | 1 | 3 | 2 | 12 | 1 |
| PA_05 | 14.62 | 6.27 | 64.16718 | 4 | 2.331738 | 2 | 3 | 1 | 1 | 3 | 2 | 12 | 1 |
| PA_06 | 11.92 | 4.55 | 37.9652 | 2 | 2.61978 | 3 | 3 | 3 | 1 | 3 | 2 | 11 | 1 |
| PA_07 | 15.72 | 6.29 | 69.21516 | 4 | 2.499205 | 2 | 2 | 3 | 1 | 3 | 2 | 8 | 1 |
| PA_08 | 14.97 | 5.07 | 53.12853 | 3 | 2.952663 | 3 | 3 | 2 | 1 | 3 | 2 | 11 | 1 |
| PA_09 | 17.09 | 7.34 | 87.80842 | 4 | 2.328338 | 2 | 2 | 3 | 1 | 3 | 2 | 9 | 1 |
| PA_10 | 13.95 | 6.42 | 62.6913 | 4 | 2.172897 | 2 | 2 | 3 | 1 | 3 | 2 | 9 | 1 |
| PA_11 | 11.15 | 4.49 | 35.04445 | 2 | 2.483296 | 2 | 3 | 2 | 1 | 3 | 2 | 11 | 1 |
| PA_12 | 10.28 | 5.02 | 36.12392 | 2 | 2.047809 | 2 | 3 | 2 | 1 | 3 | 2 | 10 | 1 |
| PA_13 | 16.17 | 7.77 | 87.94863 | 4 | 2.081081 | 2 | 3 | 2 | 1 | 3 | 2 | 12 | 1 |
| PA_14 | 8.82 | 3.96 | 24.44904 | 2 | 2.227273 | 2 | 2 | 3 | 1 | 3 | 2 | 9 | 1 |
| PA_15 | 8.33 | 5.15 | 30.02965 | 2 | 1.617476 | 1 | 5 | 1 | 1 | 3 | 2 | 9 | 1 |
| SD_01 | 10.38 | 4.35 | 31.6071 | 2 | 2.386207 | 2 | 2 | 2 | 1 | 2 | 2 | 12 | 1 |
| SD_02 | 10.93 | 4.16 | 31.82816 | 2 | 2.627404 | 3 | 2 | 2 | 1 | 2 | 2 | 11 | 1 |
| SD_03 | 10.75 | 3.85 | 28.97125 | 2 | 2.792208 | 3 | 2 | 2 | 1 | 2 | 1 | 12 | 1 |
| SD_04 | 14.7 | 6.04 | 62.1516 | 4 | 2.433775 | 2 | 3 | 3 | 1 | 2 | 2 | 11 | 1 |
| SD_05 | 9.86 | 3.99 | 27.53898 | 2 | 2.471178 | 2 | 2 | 2 | 1 | 3 | 2 | 9 | 1 |
| SD_06 | 11.44 | 5.71 | 45.72568 | 3 | 2.003503 | 2 | 1 | 3 | 2 | 2 | 1 | 11 | 1 |
| SD_07 | 9.41 | 4.02 | 26.47974 | 2 | 2.340796 | 2 | 2 | 2 | 1 | 2 | 1 | 10 | 1 |
| SD_08 | 12.73 | 4.24 | 37.78264 | 2 | 3.002358 | 4 | 2 | 1 | 1 | 2 | 2 | 12 | 1 |
| SD_09 | 11.43 | 5.22 | 41.76522 | 3 | 2.189655 | 2 | 2 | 2 | 1 | 2 | 2 | 11 | 1 |
| SD_10 | 11.87 | 4.39 | 36.47651 | 2 | 2.703872 | 3 | 2 | 2 | 1 | 2 | 2 | 12 | 1 |
| SD_11 | 11.46 | 5.39 | 43.23858 | 3 | 2.12616 | 2 | 2 | 2 | 1 | 3 | 1 | 11 | 1 |
| SD_12 | 9.93 | 4.41 | 30.65391 | 2 | 2.251701 | 2 | 2 | 2 | 1 | 2 | 2 | 12 | 1 |
| SD_13 | 16.93 | 6.85 | 81.17935 | 4 | 2.471533 | 2 | 2 | 2 | 1 | 1 | 2 | 9 | 1 |
| SD_14 | 10.25 | 5.05 | 36.23375 | 2 | 2.029703 | 2 | 2 | 2 | 1 | 3 | 1 | 12 | 1 |
| SD_15 | 14.87 | 6.41 | 66.72169 | 4 | 2.319813 | 2 | 3 | 2 | 1 | 2 | 2 | 11 | 1 |
| DY_01 | 10.73 | 4.2 | 31.5462 | 2 | 2.554762 | 3 | 3 | 3 | 1 | 3 | 1 | 13 | 3 |
| DY_02 | 10.03 | 3.97 | 27.87337 | 2 | 2.526448 | 3 | 3 | 1 | 1 | 3 | 3 | 15 | 3 |
| DY_03 | 8.23 | 2.97 | 17.11017 | 1 | 2.771044 | 3 | 3 | 1 | 1 | 3 | 3 | 13 | 3 |
| DY_04 | 7.07 | 2.5 | 12.3725 | 1 | 2.828 | 3 | 3 | 1 | 1 | 3 | 1 | 12 | 3 |
| DY_05 | 9.73 | 3.57 | 24.31527 | 2 | 2.72549 | 3 | 3 | 1 | 1 | 3 | 1 | 13 | 3 |
| DY_06 | 7.93 | 2.83 | 15.70933 | 1 | 2.80212 | 3 | 3 | 1 | 1 | 3 | 1 | 13 | 3 |
| DY_07 | 9.3 | 3.61 | 23.5011 | 2 | 2.576177 | 3 | 3 | 1 | 1 | 3 | 1 | 14 | 3 |
| DY_08 | 7.17 | 3.53 | 17.71707 | 1 | 2.031161 | 2 | 3 | 1 | 2 | 3 | 1 | 12 | 3 |
| DY_09 | 9.05 | 3.66 | 23.1861 | 2 | 2.472678 | 2 | 3 | 3 | 1 | 3 | 1 | 11 | 3 |
| DY_10 | 12.5 | 4.74 | 41.475 | 3 | 2.637131 | 3 | 3 | 3 | 1 | 3 | 1 | 13 | 3 |
| DY_11 | 8.72 | 3.62 | 22.09648 | 2 | 2.40884 | 2 | 4 | 3 | 2 | 3 | 1 | 12 | 3 |
| DY_12 | 10.18 | 4.16 | 29.64416 | 2 | 2.447115 | 2 | 3 | 3 | 2 | 3 | 3 | 13 | 3 |
| DY_13 | 11.32 | 4.46 | 35.34104 | 2 | 2.538117 | 3 | 3 | 3 | 1 | 3 | 1 | 13 | 3 |
| DY_14 | 10.3 | 3.92 | 28.2632 | 2 | 2.627551 | 3 | 4 | 3 | 1 | 3 | 1 | 14 | 3 |
| DY_15 | 10.38 | 3.96 | 28.77336 | 2 | 2.621212 | 3 | 4 | 3 | 1 | 3 | 3 | 14 | 3 |
| YH_01 | 11.51 | 5.45 | 43.91065 | 3 | 2.111927 | 2 | 3 | 2 | 1 | 2 | 2 | 7 | 2 |
| YH_02 | 12.72 | 5.56 | 49.50624 | 3 | 2.28777 | 2 | 2 | 2 | 1 | 2 | 2 | 8 | 1 |
| YH_03 | 12.67 | 6.56 | 58.18064 | 3 | 1.931402 | 1 | 2 | 2 | 2 | 2 | 1 | 11 | 1 |
| YH_04 | 5.03 | 2.04 | 7.18284 | 1 | 2.465686 | 2 | 2 | 2 | 1 | 3 | 2 | 7 | 1 |
| YH_05 | 14.93 | 6.42 | 67.09542 | 4 | 2.325545 | 2 | 2 | 2 | 1 | 2 | 2 | 10 | 2 |
| YH_06 | 13.86 | 5.99 | 58.11498 | 3 | 2.313856 | 2 | 2 | 2 | 1 | 1 | 2 | 10 | 2 |
| YH_07 | 8.73 | 4.59 | 28.04949 | 2 | 1.901961 | 1 | 2 | 1 | 2 | 3 | 2 | 11 | 2 |
| YH_08 | 8.53 | 4.73 | 28.24283 | 2 | 1.803383 | 1 | 2 | 1 | 2 | 3 | 2 | 9 | 1 |
| YH_09 | 7.92 | 3.47 | 19.23768 | 1 | 2.282421 | 2 | 2 | 1 | 3 | 3 | 2 | 10 | 1 |
| YH_10 | 9.08 | 4.05 | 25.7418 | 2 | 2.241975 | 2 | 2 | 1 | 1 | 3 | 2 | 7 | 1 |
| YH_11 | 9.29 | 4.65 | 30.23895 | 2 | 1.997849 | 1 | 2 | 1 | 2 | 3 | 2 | 7 | 1 |
| YH_12 | 8.67 | 3.84 | 23.30496 | 2 | 2.257813 | 2 | 2 | 1 | 3 | 2 | 2 | 11 | 1 |
| YH_13 | 6.8 | 3.49 | 16.6124 | 1 | 1.948424 | 1 | 1 | 1 | 3 | 3 | 2 | 9 | 1 |
| YH_14 | 6.16 | 2.86 | 12.33232 | 1 | 2.153846 | 2 | 1 | 1 | 3 | 3 | 2 | 10 | 1 |
| YH_15 | 9.44 | 3.99 | 26.36592 | 2 | 2.365915 | 2 | 3 | 2 | 1 | 3 | 2 | 11 | 1 |
| SQ_01 | 7.58 | 3.63 | 19.26078 | 1 | 2.088154 | 2 | 4 | 1 | 2 | 3 | 1 | 8 | 3 |
| SQ_02 | 7.48 | 3.18 | 16.65048 | 1 | 2.352201 | 2 | 4 | 1 | 2 | 3 | 1 | 8 | 3 |
| SQ_03 | 7.99 | 3.46 | 19.35178 | 1 | 2.309249 | 2 | 4 | 1 | 2 | 3 | 1 | 7 | 3 |
| SQ_04 | 9.4 | 2.91 | 19.1478 | 1 | 3.230241 | 4 | 4 | 1 | 2 | 3 | 1 | 9 | 3 |
| SQ_05 | 7.68 | 3.59 | 19.29984 | 1 | 2.139276 | 2 | 4 | 1 | 2 | 3 | 1 | 9 | 3 |
| SQ_06 | 8.07 | 3.48 | 19.65852 | 1 | 2.318966 | 2 | 4 | 1 | 2 | 3 | 1 | 8 | 3 |
| SQ_07 | 6.26 | 3.61 | 15.81902 | 1 | 1.734072 | 1 | 4 | 1 | 2 | 3 | 1 | 7 | 3 |
| SQ_08 | 6.01 | 2.67 | 11.23269 | 1 | 2.250936 | 2 | 4 | 1 | 2 | 3 | 1 | 8 | 3 |
| SQ_09 | 9.76 | 5.07 | 34.63824 | 2 | 1.925049 | 1 | 4 | 2 | 2 | 3 | 1 | 11 | 3 |
| SQ_10 | 5.13 | 2.14 | 7.68474 | 1 | 2.397196 | 2 | 4 | 1 | 2 | 3 | 1 | 7 | 3 |
| SQ_11 | 6.57 | 2.84 | 13.06116 | 1 | 2.31338 | 2 | 4 | 1 | 2 | 3 | 1 | 9 | 3 |
| SQ_12 | 6.96 | 3.75 | 18.27 | 1 | 1.856 | 1 | 4 | 1 | 2 | 3 | 1 | 9 | 3 |
| SQ_13 | 6.86 | 2.39 | 11.47678 | 1 | 2.870293 | 3 | 4 | 1 | 2 | 3 | 1 | 8 | 3 |
| SQ_14 | 6.81 | 2.53 | 12.06051 | 1 | 2.6917 | 3 | 4 | 1 | 2 | 3 | 1 | 8 | 3 |
| SQ_15 | 8.01 | 3.99 | 22.37193 | 2 | 2.007519 | 2 | 4 | 1 | 2 | 3 | 1 | 9 | 3 |
| HK_01 | 15.95 | 5.58 | 62.3007 | 4 | 2.858423 | 3 | 2 | 1 | 1 | 1 | 3 | 11 | 1 |
| HK_02 | 12.85 | 4.62 | 41.5569 | 3 | 2.781385 | 3 | 2 | 1 | 1 | 1 | 3 | 12 | 1 |
| HK_03 | 15.07 | 6.69 | 70.57281 | 4 | 2.252616 | 2 | 2 | 1 | 1 | 1 | 3 | 13 | 1 |
| HK_04 | 11.18 | 5.23 | 40.92998 | 3 | 2.137667 | 2 | 2 | 1 | 1 | 1 | 3 | 11 | 1 |
| HK_05 | 13.69 | 5.7 | 54.6231 | 3 | 2.401754 | 2 | 2 | 1 | 1 | 1 | 3 | 14 | 1 |
| HK_06 | 11.15 | 5 | 39.025 | 2 | 2.23 | 2 | 2 | 1 | 1 | 2 | 2 | 12 | 1 |
| HK_07 | 13.65 | 6.32 | 60.3876 | 4 | 2.15981 | 2 | 2 | 1 | 1 | 1 | 3 | 13 | 1 |
| HK_08 | 11.59 | 4.76 | 38.61788 | 2 | 2.434874 | 2 | 2 | 1 | 1 | 2 | 3 | 12 | 1 |
| HK_09 | 15.22 | 6.27 | 66.80058 | 4 | 2.427432 | 2 | 2 | 1 | 1 | 1 | 3 | 13 | 1 |
| HK_10 | 14.43 | 6.04 | 61.01004 | 4 | 2.389073 | 2 | 2 | 1 | 1 | 1 | 3 | 12 | 1 |
| HK_11 | 13.58 | 4.47 | 42.49182 | 3 | 3.038031 | 4 | 2 | 1 | 1 | 1 | 3 | 13 | 1 |
| HK_12 | 17.28 | 7.85 | 94.9536 | 4 | 2.201274 | 2 | 2 | 1 | 1 | 1 | 3 | 13 | 1 |
| HK_13 | 16.05 | 6.11 | 68.64585 | 4 | 2.626841 | 3 | 2 | 1 | 1 | 1 | 3 | 12 | 1 |
| HK_14 | 12.8 | 4.51 | 40.4096 | 3 | 2.838137 | 3 | 2 | 1 | 1 | 1 | 3 | 12 | 1 |
| HK_15 | 8.58 | 4.32 | 25.94592 | 2 | 1.986111 | 1 | 2 | 1 | 1 | 1 | 3 | 13 | 1 |
| DL_01 | 9.49 | 4.31 | 28.63133 | 2 | 2.201856 | 2 | 2 | 1 | 1 | 2 | 1 | 10 | 2 |
| DL_02 | 7.17 | 4.42 | 22.18398 | 2 | 1.622172 | 1 | 2 | 1 | 3 | 2 | 1 | 9 | 2 |
| DL_03 | 10.47 | 4.51 | 33.05379 | 2 | 2.321508 | 2 | 2 | 1 | 1 | 2 | 1 | 11 | 2 |
| DL_04 | 11.42 | 5.13 | 41.00922 | 3 | 2.226121 | 2 | 2 | 1 | 1 | 2 | 1 | 10 | 2 |
| DL_05 | 11.36 | 4.92 | 39.12384 | 2 | 2.308943 | 2 | 2 | 1 | 1 | 2 | 1 | 11 | 2 |
| DL_06 | 9.13 | 4.31 | 27.54521 | 2 | 2.118329 | 2 | 2 | 1 | 1 | 2 | 1 | 10 | 2 |
| DL_07 | 14.13 | 6.56 | 64.88496 | 4 | 2.153963 | 2 | 2 | 1 | 1 | 2 | 1 | 11 | 2 |
| DL_08 | 7.37 | 3.13 | 16.14767 | 1 | 2.354633 | 2 | 2 | 1 | 1 | 2 | 1 | 7 | 2 |
| DL_09 | 8.27 | 3.25 | 18.81425 | 1 | 2.544615 | 3 | 2 | 1 | 1 | 2 | 1 | 10 | 2 |
| DL_10 | 9.27 | 4.51 | 29.26539 | 2 | 2.055432 | 2 | 2 | 1 | 1 | 2 | 1 | 10 | 2 |
| DL_11 | 8.73 | 5.02 | 30.67722 | 2 | 1.739044 | 1 | 2 | 1 | 1 | 2 | 1 | 11 | 2 |
| DL_12 | 12.31 | 4.92 | 42.39564 | 3 | 2.502033 | 3 | 2 | 1 | 1 | 2 | 1 | 11 | 2 |
| DL_13 | 12.13 | 6.11 | 51.88001 | 3 | 1.98527 | 1 | 2 | 1 | 1 | 2 | 1 | 10 | 2 |
| DL_14 | 8.26 | 4.26 | 24.63132 | 2 | 1.938967 | 1 | 2 | 1 | 1 | 2 | 1 | 12 | 2 |
| DL_15 | 12.74 | 5.19 | 46.28442 | 3 | 2.454721 | 2 | 2 | 1 | 1 | 2 | 1 | 11 | 2 |

**Table S3 Ancient tea tree whole genome resequencing data output statistics**

| Sample  Name | Number data | Raw Bases (G) | Clean Reads | Raw Bases (G) | Q20  (%) | Q30  (%) | GC  (%) | Clean/Raw (%) | clean data | Map reads  Rate (%) | Sequencing  depth | Coverage  Rate (%) |
| --- | --- | --- | --- | --- | --- | --- | --- | --- | --- | --- | --- | --- |
| DL_01 | 306321920 | 45.95 | 291472936 | 43.72 | 96.2 | 88.2 | 40.02 | 95.15 | 284531934 | 95.31 | 11.9 | 72.1 |
| DL_02 | 290059132 | 43.51 | 278981368 | 41.85 | 96.73 | 89.3 | 39.4 | 96.18 | 277081613 | 96.99 | 11.6 | 71.84 |
| DL_03 | 306004836 | 45.9 | 294067298 | 44.11 | 96.47 | 88.6 | 39.63 | 96.1 | 292882237 | 97.2 | 12.2 | 72.66 |
| DL_04 | 298698422 | 44.8 | 286399960 | 42.96 | 96.42 | 88.5 | 39.92 | 95.88 | 282289717 | 96.16 | 11.7 | 72.54 |
| DL_05 | 216097732 | 32.41 | 203394118 | 30.51 | 95.81 | 87.5 | 41.52 | 94.12 | 184234572 | 88.7 | 7.69 | 69.24 |
| DL_06 | 302280768 | 45.34 | 290525796 | 43.58 | 96.59 | 89 | 39.65 | 96.11 | 290337703 | 97.47 | 12.3 | 72.69 |
| DL_07 | 278901770 | 41.84 | 268139592 | 40.22 | 96.49 | 88.7 | 39.62 | 96.14 | 268252737 | 97.59 | 11.4 | 72.44 |
| DL_08 | 304400140 | 45.66 | 290161438 | 43.52 | 95.9 | 87.4 | 39.89 | 95.32 | 287461125 | 96.67 | 12 | 72.73 |
| DL_09 | 300830528 | 45.12 | 286089382 | 42.91 | 96.07 | 87.9 | 39.96 | 95.1 | 280655703 | 95.85 | 11.6 | 71.64 |
| DL_10 | 265001426 | 39.75 | 252262304 | 37.84 | 95.89 | 87.4 | 39.77 | 95.19 | 250788374 | 96.99 | 10.5 | 72 |
| DL_11 | 275265346 | 41.29 | 262500008 | 39.38 | 96.22 | 88.3 | 40.22 | 95.36 | 255148109 | 95.05 | 10.5 | 70.55 |
| DL_12 | 288016838 | 43.2 | 274542240 | 41.18 | 95.96 | 87.5 | 39.64 | 95.32 | 272246789 | 96.89 | 11.3 | 71.99 |
| DL_13 | 323938154 | 48.59 | 308834664 | 46.33 | 96.02 | 87.7 | 40.3 | 95.34 | 297833064 | 94.17 | 12.5 | 72.76 |
| DL_14 | 285693834 | 42.85 | 266585454 | 39.99 | 94.78 | 85.2 | 40.06 | 93.31 | 251431781 | 92.17 | 10.5 | 71.68 |
| DL_15 | 253126568 | 37.97 | 234521254 | 35.18 | 94.35 | 84.1 | 40.11 | 92.65 | 230201809 | 95.9 | 9.55 | 71.62 |
| DY_01 | 296307762 | 44.45 | 283257138 | 42.49 | 96.24 | 88.1 | 38.88 | 95.6 | 284891897 | 98.98 | 12.7 | 78.85 |
| DY_02 | 233954208 | 35.09 | 223881514 | 33.58 | 96.31 | 88.3 | 38.49 | 95.69 | 225837509 | 99.1 | 10.1 | 78.42 |
| DY_03 | 314332984 | 47.15 | 301750896 | 45.26 | 96.59 | 89 | 39.08 | 96 | 303374791 | 98.88 | 13.4 | 78.74 |
| DY_04 | 288135276 | 43.22 | 272975288 | 40.95 | 95.91 | 87.4 | 38.58 | 94.74 | 274277091 | 98.81 | 12.3 | 79.34 |
| DY_05 | 281066508 | 42.16 | 266091364 | 39.91 | 95.76 | 87 | 38.52 | 94.67 | 267537054 | 98.99 | 11.9 | 78.91 |
| DY_06 | 292244850 | 43.84 | 273677622 | 41.05 | 95.02 | 85.5 | 38.82 | 93.65 | 274460620 | 98.74 | 12.3 | 78.9 |
| DY_07 | 305432718 | 45.81 | 290594444 | 43.59 | 96.08 | 87.8 | 38.66 | 95.14 | 292675857 | 98.98 | 13 | 79.41 |
| DY_08 | 271048842 | 40.66 | 254822878 | 38.22 | 95.38 | 86.2 | 38.68 | 94.01 | 255661671 | 98.75 | 11.4 | 79.4 |
| DY_09 | 271604350 | 40.74 | 255744096 | 38.36 | 95.58 | 86.6 | 38.7 | 94.16 | 257408305 | 99.05 | 11.5 | 78.5 |
| DY_10 | 237832614 | 35.67 | 221926630 | 33.29 | 95.19 | 85.8 | 39.33 | 93.31 | 213970176 | 94.55 | 9.45 | 78.12 |
| DY_11 | 244955064 | 36.74 | 229236886 | 34.39 | 95.68 | 87 | 39.14 | 93.58 | 219911002 | 94.34 | 9.78 | 77.46 |
| DY_12 | 236556174 | 35.48 | 220629116 | 33.09 | 95.02 | 85.6 | 39.41 | 93.27 | 212051702 | 94.55 | 9.42 | 77.92 |
| DY_13 | 243094046 | 36.46 | 228381344 | 34.26 | 95.23 | 85.9 | 38.8 | 93.95 | 225279702 | 96.97 | 10.1 | 78.45 |
| DY_14 | 245326924 | 36.8 | 228595388 | 34.29 | 94.81 | 84.9 | 38.64 | 93.18 | 229455600 | 98.68 | 10.2 | 78.77 |
| DY_15 | 285064810 | 42.76 | 268269236 | 40.24 | 95.41 | 86.3 | 38.79 | 94.11 | 264593078 | 96.96 | 11.8 | 78.46 |
| HK_01 | 241214892 | 36.18 | 219512344 | 32.93 | 93.73 | 83.1 | 42.26 | 91 | 178222630 | 79.92 | 7.8 | 74.91 |
| HK_02 | 245942328 | 36.89 | 230825424 | 34.62 | 95.53 | 86.8 | 41.87 | 93.85 | 228977621 | 85.81 | 10 | 76.81 |
| HK_03 | 285166586 | 42.77 | 271045002 | 40.66 | 95.9 | 87.4 | 40.3 | 95.05 | 252729504 | 91.5 | 11 | 76.96 |
| HK_04 | 314197284 | 47.13 | 302719008 | 45.41 | 96.9 | 89.5 | 39.93 | 96.35 | 289891621 | 94.03 | 12.2 | 76.73 |
| HK_05 | 312563418 | 46.88 | 296807392 | 44.52 | 95.96 | 87.7 | 41.58 | 94.96 | 258025451 | 85.45 | 11.2 | 76.73 |
| HK_06 | 307691432 | 46.15 | 292996918 | 43.95 | 95.95 | 87.5 | 40.18 | 95.22 | 280081153 | 93.79 | 12.1 | 77.07 |
| HK_07 | 272931388 | 40.94 | 260286680 | 39.04 | 96.32 | 88.1 | 41.28 | 95.37 | 229114532 | 86.46 | 10 | 76.17 |
| HK_08 | 288360658 | 43.25 | 274007796 | 41.1 | 96.11 | 87.9 | 39.66 | 95.02 | 261231986 | 93.56 | 11.3 | 76.55 |
| HK_09 | 300193420 | 45.03 | 285751200 | 42.86 | 96.42 | 88.5 | 42.24 | 95.19 | 235434921 | 81.12 | 9.96 | 75.79 |
| HK_10 | 313451814 | 47.02 | 299462982 | 44.92 | 96.42 | 88.5 | 39.81 | 95.54 | 290157182 | 95.13 | 12.2 | 76.59 |
| HK_11 | 299901128 | 44.99 | 286157034 | 42.92 | 96.17 | 87.8 | 39.41 | 95.42 | 281578948 | 96.5 | 12.3 | 77.34 |
| HK_12 | 252802480 | 37.92 | 238458856 | 35.77 | 95.73 | 87 | 40.27 | 94.33 | 223871625 | 92.27 | 9.44 | 75.68 |
| HK_13 | 315030372 | 47.25 | 297862008 | 44.68 | 96.25 | 88.6 | 41.7 | 94.55 | 259783215 | 85.69 | 11.3 | 76.12 |
| HK_14 | 326131740 | 48.92 | 306495920 | 45.97 | 96.13 | 88.3 | 42.12 | 93.98 | 260727290 | 83.66 | 11.3 | 76.32 |
| HK_15 | 301527724 | 45.23 | 276690496 | 41.5 | 95.24 | 86.5 | 46 | 91.76 | 178241176 | 63.64 | 7.59 | 73.36 |
| PA_01 | 307155996 | 46.07 | 291875304 | 43.78 | 96.24 | 88.3 | 41.07 | 95.03 | 267139763 | 89.54 | 11.3 | 71.48 |
| PA_02 | 276030914 | 41.4 | 262497574 | 39.37 | 95.95 | 87.2 | 39.54 | 95.1 | 260281352 | 96.65 | 11.3 | 72.89 |
| PA_03 | 286294546 | 42.94 | 273895578 | 41.08 | 96.28 | 88.1 | 39.52 | 95.67 | 272334867 | 96.98 | 11.7 | 72.65 |
| PA_04 | 277251338 | 41.59 | 265136996 | 39.77 | 96.31 | 88.3 | 39.67 | 95.63 | 264367866 | 97.28 | 11.2 | 72.16 |
| PA_05 | 308246410 | 46.24 | 292083190 | 43.81 | 95.88 | 87.2 | 39.72 | 94.76 | 288557710 | 96.46 | 12.3 | 72.35 |
| PA_06 | 336539422 | 50.48 | 316555390 | 47.48 | 95.52 | 86.5 | 40.15 | 94.06 | 299655108 | 92.5 | 12.8 | 72.99 |
| PA_07 | 305583982 | 45.84 | 292994052 | 43.95 | 96.56 | 89 | 39.63 | 95.88 | 289297938 | 96.4 | 12.3 | 72.35 |
| PA_08 | 325280746 | 48.79 | 309930212 | 46.49 | 96.16 | 88.1 | 40.95 | 95.28 | 286561704 | 90.37 | 12.2 | 72.58 |
| PA_09 | 232443862 | 34.87 | 219350674 | 32.9 | 95.74 | 87 | 40.19 | 94.37 | 207928404 | 92.65 | 8.95 | 70.03 |
| PA_10 | 305075780 | 45.76 | 288023718 | 43.2 | 95.97 | 87.7 | 40.02 | 94.41 | 273059379 | 92.6 | 11.7 | 72.5 |
| PA_11 | 289949286 | 43.49 | 276080964 | 41.41 | 96.2 | 87.9 | 39.95 | 95.22 | 262676695 | 93.05 | 11 | 71.22 |
| PA_12 | 322712444 | 48.41 | 306258138 | 45.94 | 96.42 | 88.4 | 38.78 | 94.9 | 285569455 | 91.15 | 12.1 | 71.93 |
| PA_13 | 297975704 | 44.7 | 282586104 | 42.39 | 96.03 | 87.4 | 39.83 | 94.84 | 271603408 | 93.9 | 11.6 | 71.93 |
| PA_14 | 293253026 | 43.99 | 278883080 | 41.83 | 95.98 | 87.2 | 39.76 | 95.1 | 276645702 | 96.77 | 11.9 | 72.61 |
| PA_15 | 291091156 | 43.66 | 276330742 | 41.45 | 96.35 | 88.3 | 40.1 | 94.93 | 267824168 | 94.7 | 11.5 | 73.71 |
| SD_01 | 244208054 | 36.63 | 227995104 | 34.2 | 95.01 | 85.6 | 40.49 | 93.36 | 211451346 | 90.66 | 9.14 | 71.59 |
| SD_02 | 260817674 | 39.12 | 239534260 | 35.93 | 94.54 | 84.9 | 42.7 | 91.84 | 189198430 | 77.44 | 8.17 | 70.33 |
| SD_03 | 262479334 | 39.37 | 248706162 | 37.31 | 95.98 | 87.4 | 40.6 | 94.75 | 222490106 | 87.46 | 9.57 | 71.79 |
| SD_04 | 230012346 | 34.5 | 209925208 | 31.49 | 94.27 | 83.8 | 40.1 | 91.27 | 202896608 | 94.51 | 8.72 | 71.8 |
| SD_05 | 283222212 | 42.48 | 267571346 | 40.14 | 96.06 | 87.7 | 42.15 | 94.47 | 217410734 | 79.61 | 9.37 | 71.18 |
| SD_06 | 262837304 | 39.43 | 250693992 | 37.6 | 96.29 | 88.1 | 40.41 | 95.38 | 233218703 | 90.88 | 10 | 71.87 |
| SD_07 | 206812846 | 31.02 | 194797462 | 29.22 | 95.55 | 86.6 | 40.23 | 94.19 | 178139461 | 89.37 | 7.64 | 70.58 |
| SD_08 | 303644260 | 45.55 | 287950132 | 43.19 | 96.09 | 88 | 40.17 | 94.83 | 258106987 | 87.7 | 11 | 71.31 |
| SD_09 | 301452598 | 45.22 | 281918246 | 42.29 | 95.6 | 86.8 | 42.25 | 93.52 | 232913001 | 80.86 | 10.1 | 71.98 |
| SD_10 | 331911266 | 49.79 | 317165704 | 47.57 | 96.36 | 88.5 | 39.75 | 95.56 | 307599214 | 94.71 | 13.2 | 73.2 |
| SD_11 | 256914966 | 38.54 | 242824248 | 36.42 | 95.84 | 87.3 | 40.02 | 94.52 | 228630137 | 91.99 | 9.87 | 72.04 |
| SD_12 | 274732848 | 41.21 | 254881966 | 38.23 | 95.14 | 85.6 | 42.79 | 92.77 | 217162642 | 83.44 | 9.37 | 71.27 |
| SD_13 | 246184668 | 36.93 | 231456088 | 34.72 | 95.36 | 85.8 | 39.76 | 94.02 | 227817295 | 96.13 | 9.84 | 72.01 |
| SD_14 | 238222078 | 35.73 | 224512798 | 33.68 | 95.62 | 86.5 | 39.8 | 94.25 | 218448534 | 95.01 | 9.41 | 70.9 |
| SD_15 | 294403838 | 44.16 | 278011904 | 41.7 | 95.72 | 87 | 40.33 | 94.43 | 259811857 | 91.35 | 11.2 | 72.49 |
| SQ_01 | 327649634 | 49.15 | 315857000 | 47.38 | 97.1 | 90 | 39.07 | 96.4 | 316622064 | 98.65 | 14 | 79.32 |
| SQ_02 | 332144002 | 49.82 | 319264910 | 47.89 | 97.02 | 89.8 | 39.05 | 96.12 | 319942445 | 98.63 | 14.2 | 79.46 |
| SQ_03 | 312550704 | 46.88 | 300114520 | 45.02 | 96.72 | 89 | 39.31 | 96.02 | 301120881 | 98.76 | 13.3 | 79.3 |
| SQ_04 | 345707894 | 51.86 | 333774126 | 50.07 | 97.06 | 89.9 | 39.02 | 96.55 | 334567086 | 98.67 | 14.8 | 80.48 |
| SQ_05 | 287707626 | 43.16 | 272834214 | 40.93 | 96.08 | 87.8 | 39.17 | 94.83 | 272885997 | 98.53 | 11.9 | 78.43 |
| SQ_06 | 346998842 | 52.05 | 332097444 | 49.81 | 96.75 | 89.3 | 40.03 | 95.71 | 326474071 | 96.85 | 14.3 | 79.15 |
| SQ_07 | 348516746 | 52.28 | 337816598 | 50.67 | 97.29 | 90.5 | 39.7 | 96.93 | 335418007 | 97.8 | 14.6 | 79.28 |
| SQ_08 | 341919638 | 51.29 | 327186946 | 49.08 | 96.69 | 89.3 | 40.69 | 95.69 | 305808750 | 92.1 | 13.6 | 79.2 |
| SQ_09 | 355183420 | 53.28 | 343775466 | 51.57 | 97.47 | 90.9 | 39.48 | 96.79 | 339306411 | 97.14 | 15 | 79.7 |
| SQ_10 | 330877064 | 49.63 | 318807160 | 47.82 | 96.94 | 89.6 | 39.09 | 96.35 | 313894034 | 96.88 | 14 | 79.36 |
| SQ_11 | 332007228 | 49.8 | 316888640 | 47.53 | 96.25 | 88.3 | 39.46 | 95.45 | 313694786 | 97.51 | 13.7 | 78.94 |
| SQ_12 | 309555712 | 46.43 | 292860148 | 43.93 | 95.74 | 87.2 | 40.26 | 94.61 | 279056434 | 93.9 | 12.3 | 77.74 |
| SQ_13 | 301377190 | 45.21 | 287675982 | 43.15 | 96.24 | 88.3 | 39.85 | 95.45 | 274282079 | 93.89 | 12.2 | 80.66 |
| SQ_14 | 276574678 | 41.49 | 260959524 | 39.14 | 95.47 | 86.6 | 41.03 | 94.35 | 259367654 | 97.88 | 11.4 | 78.17 |
| SQ_15 | 297813166 | 44.67 | 281402308 | 42.21 | 95.57 | 86.8 | 39.1 | 94.49 | 281766868 | 98.61 | 12.4 | 78.98 |
| XS_01 | 269894186 | 40.48 | 257453776 | 38.62 | 96.19 | 87.8 | 39.77 | 95.39 | 246148181 | 93.39 | 10.5 | 72.57 |
| XS_02 | 296821306 | 44.52 | 279028614 | 41.85 | 95.1 | 86 | 39.9 | 94.01 | 277503709 | 97.16 | 11.7 | 72.25 |
| XS_03 | 288257252 | 43.24 | 271212018 | 40.68 | 96 | 87.6 | 39.66 | 94.09 | 254054880 | 91.55 | 10.8 | 72.33 |
| XS_04 | 347696404 | 52.15 | 335838440 | 50.38 | 97.36 | 90.6 | 39.9 | 96.59 | 326430324 | 94.88 | 13.7 | 72.37 |
| XS_05 | 318881778 | 47.83 | 305861066 | 45.88 | 96.67 | 89 | 40.13 | 95.92 | 291101735 | 92.98 | 12.2 | 72.11 |
| XS_06 | 294415840 | 44.16 | 280024926 | 42 | 96.31 | 88.1 | 40.04 | 95.11 | 303799738 | 95.18 | 12.8 | 72.23 |
| XS_07 | 355637266 | 53.35 | 340532470 | 51.08 | 96.81 | 89.6 | 39.89 | 95.75 | 336474358 | 96.46 | 14.1 | 72.74 |
| XS_08 | 311444802 | 46.72 | 296455424 | 44.47 | 96.19 | 87.7 | 39.89 | 95.19 | 292556858 | 96.29 | 12.4 | 72.5 |
| XS_09 | 311782364 | 46.77 | 299307214 | 44.9 | 96.85 | 89.3 | 39.49 | 96 | 294517844 | 96.09 | 12.2 | 71.81 |
| XS_10 | 279601656 | 41.94 | 268218726 | 40.23 | 96.8 | 89.2 | 39.58 | 95.93 | 264901533 | 96.31 | 11.2 | 71.84 |
| XS_11 | 292701922 | 43.91 | 280434534 | 42.07 | 96.52 | 88.3 | 39.4 | 95.81 | 280419914 | 97.51 | 11.9 | 72.41 |
| XS_12 | 338713554 | 50.81 | 326326378 | 48.95 | 97.35 | 90.7 | 39.64 | 96.34 | 325168830 | 97.23 | 13.5 | 72.28 |
| XS_13 | 294925394 | 44.24 | 279328086 | 41.9 | 96.47 | 88.3 | 39.12 | 94.71 | 281224265 | 98.15 | 11.9 | 72.29 |
| XS_14 | 304791234 | 45.72 | 292973680 | 43.95 | 96.9 | 89.4 | 39.52 | 96.12 | 293705882 | 97.82 | 12.4 | 73.18 |
| XS_15 | 297412088 | 44.61 | 286340462 | 42.95 | 97.05 | 89.8 | 39.75 | 96.28 | 285221197 | 97.25 | 11.8 | 71.71 |
| YH_01 | 307953180 | 46.19 | 295656460 | 44.35 | 96.32 | 88.3 | 39.21 | 96.01 | 294944459 | 97.77 | 12.8 | 79.88 |
| YH_02 | 317170736 | 47.58 | 305507544 | 45.83 | 97 | 89.6 | 39.57 | 96.32 | 305557195 | 97.58 | 12.9 | 72.78 |
| YH_03 | 304334408 | 45.65 | 285249450 | 42.79 | 96.56 | 88.6 | 39.44 | 93.73 | 283852007 | 97.1 | 12.1 | 72.15 |
| YH_04 | 298962694 | 44.84 | 285555568 | 42.83 | 96.31 | 88.3 | 39.67 | 95.52 | 285532279 | 97.57 | 12.2 | 72 |
| YH_05 | 327804900 | 49.17 | 314425320 | 47.16 | 97.11 | 90.1 | 39.59 | 95.92 | 312307335 | 97.38 | 13.5 | 79.49 |
| YH_06 | 276020650 | 41.4 | 261553152 | 39.23 | 95.96 | 87.2 | 39.35 | 94.76 | 261430595 | 97.95 | 11.5 | 79.01 |
| YH_07 | 312386478 | 46.86 | 298607776 | 44.79 | 96.46 | 88.3 | 39.27 | 95.59 | 295748065 | 97.58 | 12.8 | 78.32 |
| YH_08 | 331297744 | 49.69 | 316528724 | 47.48 | 96.44 | 88.2 | 39.15 | 95.54 | 314477706 | 97.88 | 13.8 | 78.8 |
| YH_09 | 305605616 | 45.84 | 293958900 | 44.09 | 96.54 | 88.1 | 39.44 | 96.19 | 291884380 | 97.83 | 12.8 | 78.86 |
| YH_10 | 350309912 | 52.55 | 334595174 | 50.19 | 96.37 | 88.4 | 39.16 | 95.51 | 334537163 | 98.43 | 14.7 | 79.04 |
| YH_11 | 296250392 | 44.44 | 281826418 | 42.27 | 95.86 | 87.1 | 38.94 | 95.13 | 280760776 | 98.09 | 12.4 | 78.59 |
| YH_12 | 232952006 | 34.94 | 220468664 | 33.07 | 95.78 | 86.8 | 39.22 | 94.64 | 230652682 | 97.78 | 10.3 | 77.96 |
| YH_13 | 278953574 | 41.84 | 264133350 | 39.62 | 96.03 | 87.4 | 39.49 | 94.69 | 261298002 | 97.02 | 11.3 | 78.46 |
| YH_14 | 320938674 | 48.14 | 308247980 | 46.24 | 96.58 | 88.7 | 40.56 | 96.05 | 308594893 | 98.6 | 13.6 | 79.56 |
| YH_15 | 289991494 | 43.5 | 275370598 | 41.31 | 95.97 | 87.5 | 41.95 | 94.96 | 275995960 | 98.59 | 12.1 | 79.34 |

**Table S4 Whole-genome SNP and INDEL distribution shown in Figure 1c and 1d**

| **Category** | **Numbers** | **Category** | **Numbers** |
| --- | --- | --- | --- |
| nonsynonymous | 90,502 | frameshift deletion | 66,742 |
| stopgain | 1,462 | rameshift insertion | 35,854 |
| stoploss | 171 | frameshift substitution | 974 |
| synonymous | 85,958 | nonframeshift deletion | 28,814 |
| downstream | 153,629 | nonframeshift insertion | 20,209 |
| exonic | 175,824 | nonframeshift substitution | 491 |
| exonic;splicing | 2,270 | stopgain | 6,035 |
| intergenic | 6,677,596 | stoploss | 265 |
| intronic | 955,080 | downstream | 403,528 |
| upstream | 114,253 | exonic | 156,734 |
| upstream;downstream | 2,793 | exonic;splicing | 2,650 |
| splicing | 925 | intergenic | 15,816,135 |
|  |  | intronic | 1,916,929 |
|  |  | splicing | 3,653 |
|  |  | upstream | 414,931 |
|  |  | upstream;downstream | 7,034 |

**Table S5 The information of different traits from the 120 ancient wild tea plants.**

|  |  | Species and varieties | Plant type | Leaf area | Leaf shape | Leaf color | Leaf apex shape | Leaf texture | Density of leaf serration | Depth of leaf serration | Number of vein pairs |
| --- | --- | --- | --- | --- | --- | --- | --- | --- | --- | --- | --- |
| Group I | XS | *C. remotiserrata* | Arbor | Large/  Extra large | Near round/  Elliptic | Medium green/  Light green | Attenuate | Medium | Sparse | Shallow | 9.8 |
|  | SD | *C. sinensis* | Arbor | Medium/ Large | Elliptic | Light green | Attenuate | Medium | Medium | Medium | 11.1 |
|  | PA | *C.quinuelocularis* | Arbor | Large/  Extra large | Elliptic | Medium green | Attenuate | Soft/Hard | Dense | Medium | 10.7 |
|  | DL | *C. taliensis* | Arbor | Small/ Medium | Elliptic/  Oblong | Medium green/  Light green | Attenuate | Soft | Sparse | Shallow | 10.6 |
| Group Ⅱ | SQ | *C. sinensis* | Shrub | Small | Elliptic | Dark green | Obtuse | Soft | Dense | Shallow | 8.2 |
| Group Ⅲ | DY | *C. sinensis* | Shrub | Small/ Medium | Oblong | Medium green | Attenuate/  Obtuse | Soft/Hard | Dense | Shallow | 15 |
|  | YH | *C. tachangensis, C. remotiserrata* | Arbor | Medium/ Large | Elliptic | Yellow green | Obtuse | Soft/Medium | Medium/Dense | Medium | 9.2 |
|  | HK | *C. assamica* | Arbor | Medium/ Large | Elliptic | Light green | Attenuate | Hard | Sparse/Medium | Deep | 12.4 |


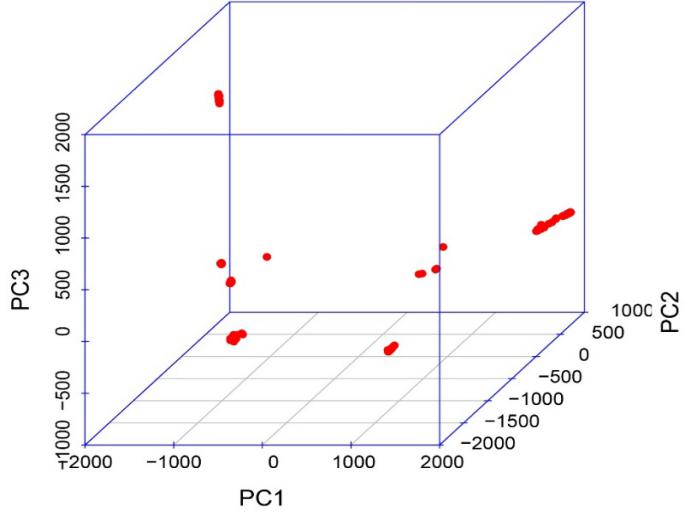


**Figure S1 Three-dimensional PCA analysis of the ancient wild tea accessions.** Red dot represents each member of eight population.


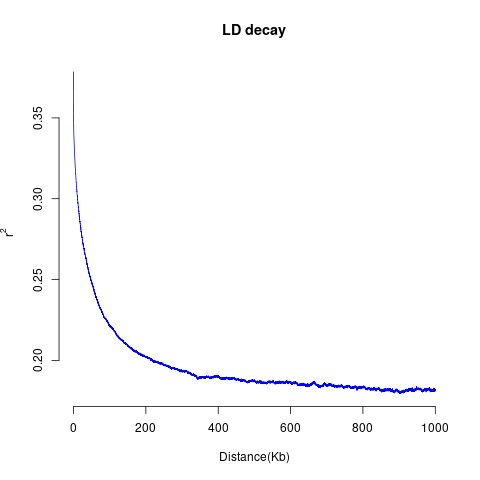


**Figure S2 LD analysis of the 120 ancient wild tea accessions.**

a b


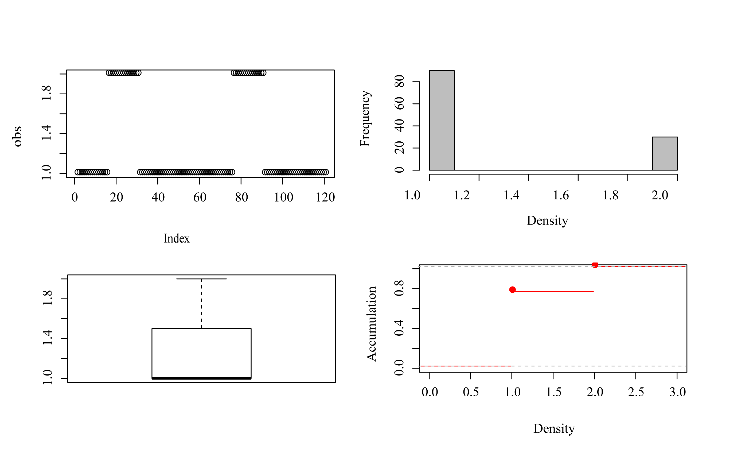

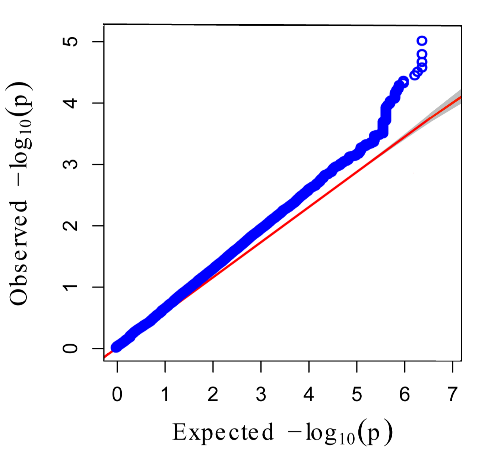


c


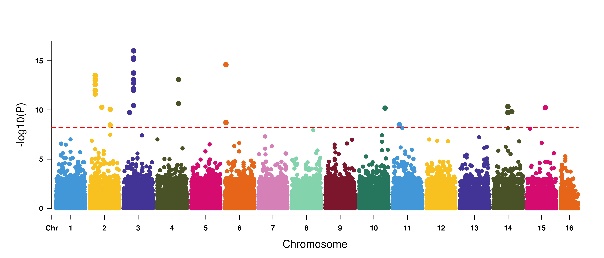


**Figure S3 GWAS analysis of plant type in 120 ancient tea accessions.** (a) GWAS analysis of plant type index from ancient tea accessions. (b) Quantile-quantile plots for 120 corresponding plant type. (c) Manhattan plots for 120 GWAS data, the significant threshold of –logP value was set at 8.2.

**a b**


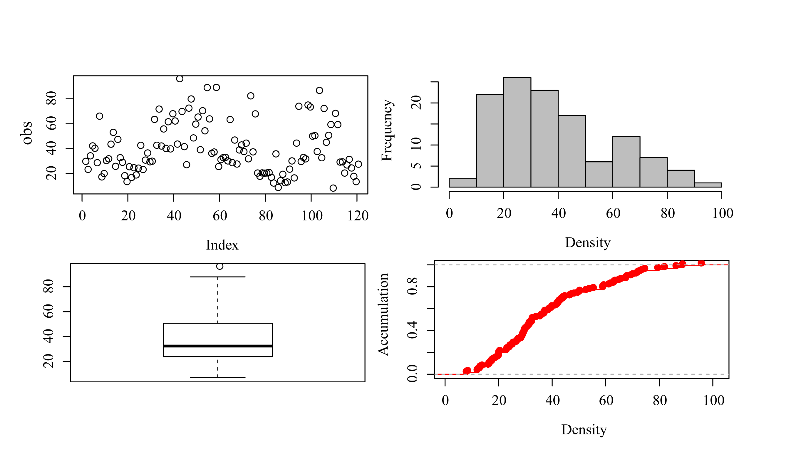

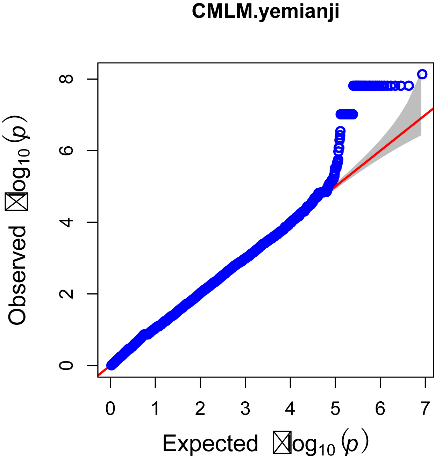


**c**


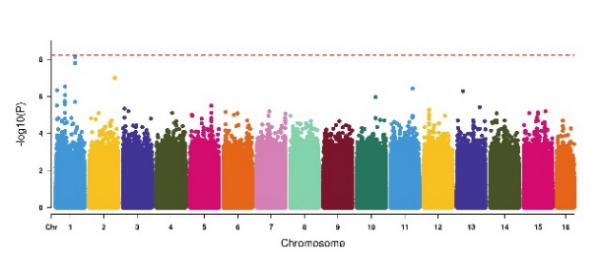


**Figure S4 GWAS analysis of leaf area in 120 ancient tea accessions.** (a) GWAS analysis of leaf area index from ancient tea accessions. (b) Quantile-quantile plots for 120 corresponding leaf area. (c) Manhattan plots for 120 GWAS data, the significant threshold of –logP value was set at 8.2.

**a b**


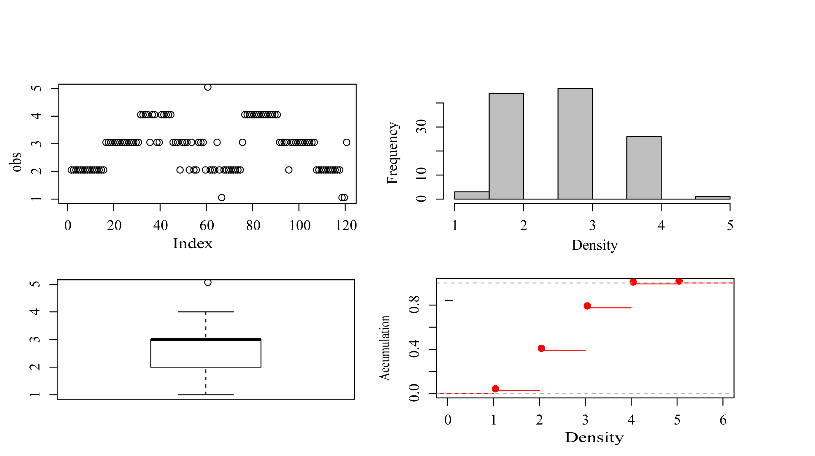

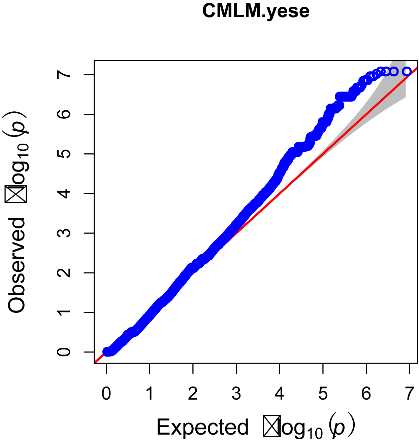


**c**


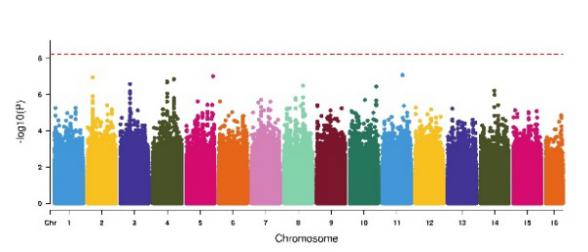


**Figure S5 GWAS analysis of leaf color in 120 ancient tea accessions.** (a) GWAS analysis of leaf color index from ancient tea accessions. (b) Quantile-quantile plots for 120 corresponding leaf color. (c) Manhattan plots for 120 GWAS data, the significant threshold of –logP value was set at 8.2.

**a b**


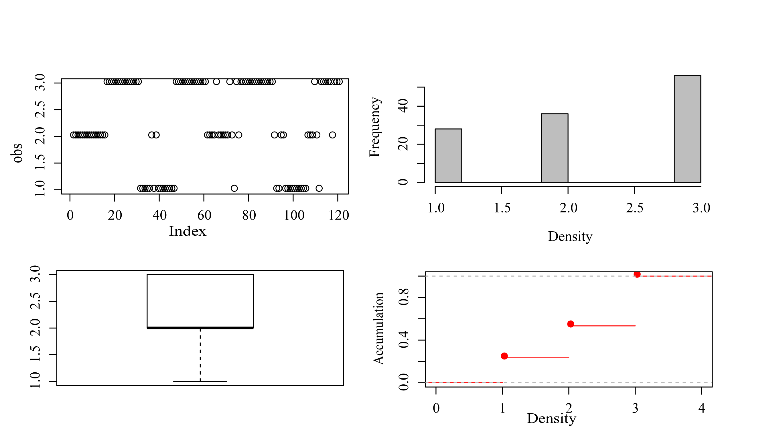

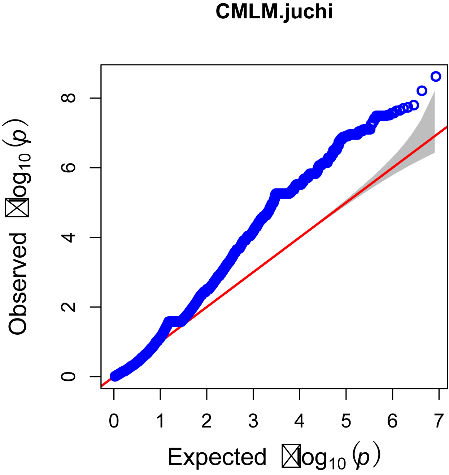


**c**


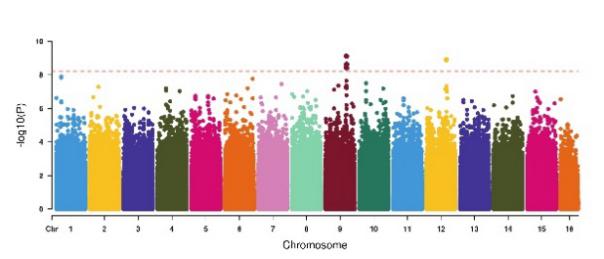


**Figure S6 GWAS analysis of the density of leaf serration in 120 ancient tea accessions.**

(a) GWAS analysis of the density of leaf serration index from ancient tea accessions. (b) Quantile-quantile plots for 120 corresponding the density of leaf serration. (c) Manhattan plots for 120 GWAS data, the significant threshold of –logP value was set at 8.2.

**a b**


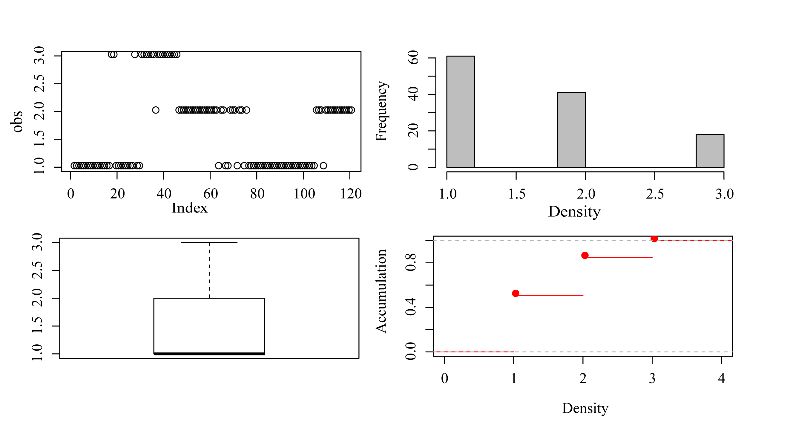

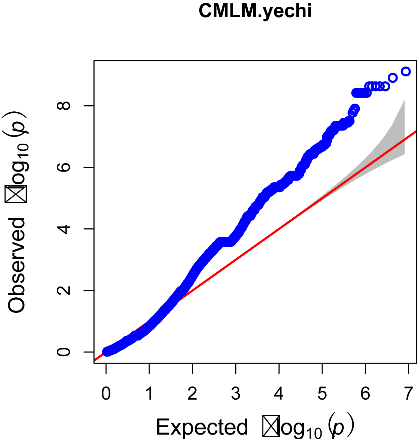


**c**


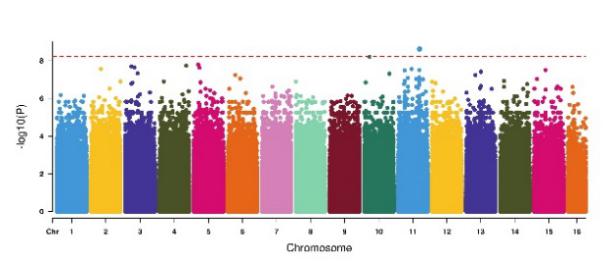


**Figure S7 GWAS analysis of the depth of leaf serration in 120 ancient tea accessions.** (a) GWAS analysis of the depth of leaf serration index from ancient tea accessions. (b) Quantile-quantile plots for 120 corresponding the depth of leaf serration. (c) Manhattan plots for 120 GWAS data, the significant threshold of –logP value was set at 8.2.

**Table S6 The information of Significant nsSNPs associated with different traits**

| Traits | Gene Locus | Exon | SNP | | Protein | | P-value | -Log10(P) |
| --- | --- | --- | --- | --- | --- | --- | --- | --- |
|  |  |  | Gene position | Codon | Protein location | Amino acid |  |  |
| Plant type | TEA012294 | exon1 | 475 | ACA-TCA | 159 | Gly-Glu | 1.99E-09 | 8.70070614 |
|  | TEA029928 | exon4 | 532 | GGG-GAG | 178 | Gly-Ser | 3.41E-09 | 8.4676573 |
| Leaf color | TEA012477 | exon20 | 4184 | ATA-ACA | 1395 | IIe-Thr | 3.64E-09 | 8.43856841 |
| Density of leaf serration | TEA025567 | exon4 | 428 | GAA-GCA | 143 | Glu-Ala | 1.26E-09 | 8.90128014 |
| Depth of leaf serration | TEA017338 | exon3 | 657 | GTC-GTT | 107 | Ser-Phe | 4.74E-08 | 8.32428906 |

**Table S7 The verification of nsSNPs associated with plant type on the chromosome-level genome**

| Traits | Gene Locus^a^ | Scaffold | Chr | Gene Locus^b^ | SNP position | Shrub (‘shuchazao’) | | Arbor | |
| --- | --- | --- | --- | --- | --- | --- | --- | --- | --- |
|  |  |  |  |  |  | Codon | Amino acid | Codon | Amino acid |
| Plant type | TEA012294/TEA029928 | Scaffold338/  Scaffold251 | Chr9/  Chr11 | CSS0013626/  CSS0001600 | 475/532 | ACA/GAG | Glu/Ser | TCA/GGG | Gly/Gly |

*Note:* a and b represent the gene locus on the Scaffold and Chromosome-level genome, respectively.

| Traits | Gene Locus^a^ | Scaffold | Chr | Gene Locus^b^ | SNP position | Dark green (‘shuchazao’) | | Light green | |
| --- | --- | --- | --- | --- | --- | --- | --- | --- | --- |
|  |  |  |  |  |  | Codon | Amino acid | Codon | Amino acid |
| Leaf color | TEA012477 | Scaffold2733 | Chr2 | CSS0020857 | 4184 | ATA | IIe | ACA | Thr |

**Table S8 The verification of nsSNPs associated with leaf color on the chromosome-level genome**

*Note:* a and b represent the gene locus on the Scaffold and Chromosome-level genome, respectively.


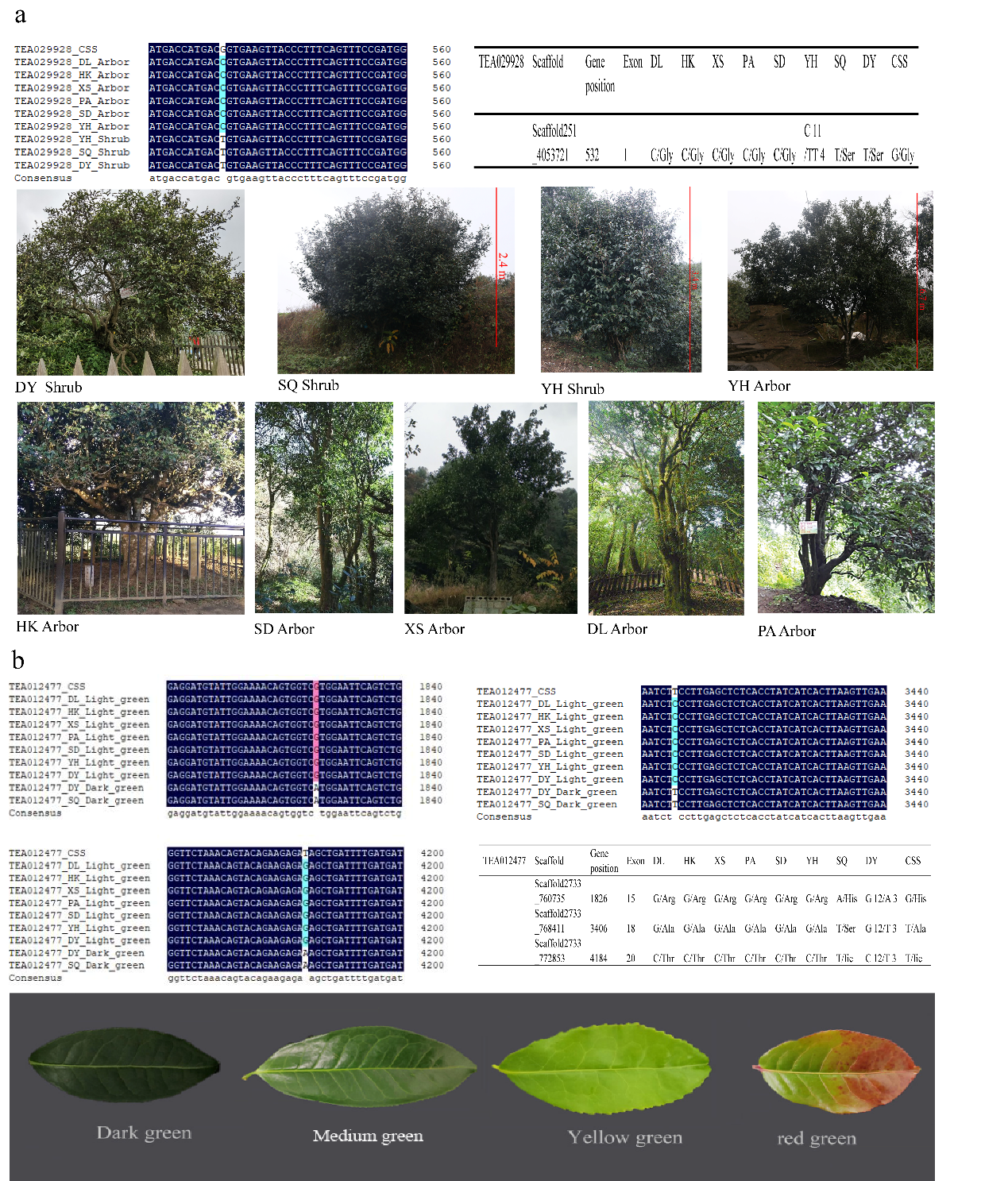


**Figure S8 The alignment of TEA012477 and TEA029928 related to leaf color and plant type, respectively.** (a) The alignment of TEA012477 related to leaf color and the distribution of nsSNP in different populations; (b) The alignment of TEA029928 related to plant type and the distribution of three nsSNPs in different populations;


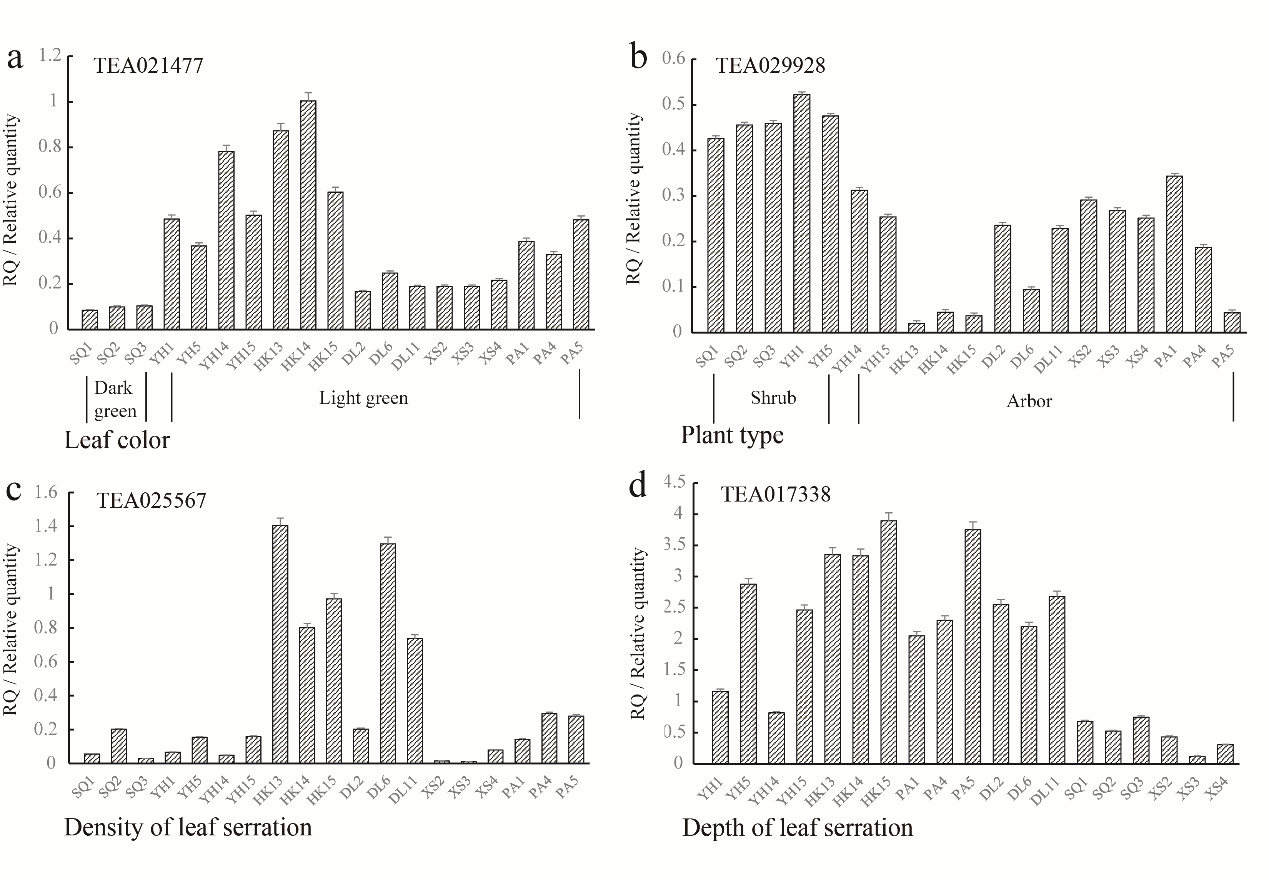


**Figure S9 The expression pattern of the genes mined using GWAS in different groups.** (a) The expression pattern of TEA021477 gene related to leaf color in different populations; (b) The expression pattern of TEA029928 gene related to plant type in different populations; (c) The expression pattern of TEA025567 gene related to density of leaf serration in different populations;(d) The expression pattern of TEA025567 gene related to density of leaf serration in different populations; (d) The expression pattern of TEA01733 genes in different populations. The expression levels relative to *GAPDH* were measured by quantitative RT-qPCR. Three biological replicates and three technical replicates were obtained for each data point.

**Table S9 Publicly accessible database number**

| Accession | Ftudy | Object  status | Bioproject  accession | Biosample  accession | Sample  name | Library  ID | Filename | Filename2 |
| --- | --- | --- | --- | --- | --- | --- | --- | --- |
| SRR14168434 | SRP313870 | new | PRJNA716079 | SAMN18590535 | DL_01 | DL_01 | DL_01_1.fq | DL_01_2.fq |
| SRR14168433 | SRP313870 | new | PRJNA716079 | SAMN18590536 | DL_02 | DL_02 | DL_02_1.fq | DL_02_2.fq |
| SRR14168427 | SRP313870 | new | PRJNA716079 | SAMN18590537 | DL_03 | DL_03 | DL_03_1.fq | DL_03_2.fq |
| SRR14168426 | SRP313870 | new | PRJNA716079 | SAMN18590538 | DL_04 | DL_04 | DL_04_1.fq | DL_04_2.fq |
| SRR14168425 | SRP313870 | new | PRJNA716079 | SAMN18590539 | DL_05 | DL_05 | DL_05_1.fq | DL_05_2.fq |
| SRR14168424 | SRP313870 | new | PRJNA716079 | SAMN18590540 | DL_06 | DL_06 | DL_06_1.fq | DL_06_2.fq |
| SRR14168423 | SRP313870 | new | PRJNA716079 | SAMN18590541 | DL_07 | DL_07 | DL_07_1.fq | DL_07_2.fq |
| SRR14168422 | SRP313870 | new | PRJNA716079 | SAMN18590542 | DL_08 | DL_08 | DL_08_1.fq | DL_08_2.fq |
| SRR14168421 | SRP313870 | new | PRJNA716079 | SAMN18590543 | DL_09 | DL_09 | DL_09_1.fq | DL_09_2.fq |
| SRR14168420 | SRP313870 | new | PRJNA716079 | SAMN18590544 | DL_10 | DL_10 | DL_10_1.fq | DL_10_2.fq |
| SRR14168432 | SRP313870 | new | PRJNA716079 | SAMN18590545 | DL_11 | DL_11 | DL_11_1.fq | DL_11_2.fq |
| SRR14168431 | SRP313870 | new | PRJNA716079 | SAMN18590546 | DL_12 | DL_12 | DL_12_1.fq | DL_12_2.fq |
| SRR14168430 | SRP313870 | new | PRJNA716079 | SAMN18590547 | DL_13 | DL_13 | DL_13_1.fq | DL_13_2.fq |
| SRR14168429 | SRP313870 | new | PRJNA716079 | SAMN18590548 | DL_14 | DL_14 | DL_14_1.fq | DL_14_2.fq |
| SRR14168428 | SRP313870 | new | PRJNA716079 | SAMN18590549 | DL_15 | DL_15 | DL_15_1.fq | DL_15_2.fq |
| SRR14179696 | SRP313870 | new | PRJNA716079 | SAMN18650255 | DY01 | DY01 | DY_01_1.fq.gz | DY_01_2.fq.gz |
| SRR14179695 | SRP313870 | new | PRJNA716079 | SAMN18650256 | DY02 | DY02 | DY_02_1.fq.gz | DY_02_2.fq.gz |
| SRR14179689 | SRP313870 | new | PRJNA716079 | SAMN18650257 | DY03 | DY03 | DY_03_1.fq.gz | DY_03_2.fq.gz |
| SRR14179688 | SRP313870 | new | PRJNA716079 | SAMN18650258 | DY04 | DY04 | DY_04_1.fq.gz | DY_04_2.fq.gz |
| SRR14179687 | SRP313870 | new | PRJNA716079 | SAMN18650259 | DY05 | DY05 | DY_05_1.fq.gz | DY_05_2.fq.gz |
| SRR14179686 | SRP313870 | new | PRJNA716079 | SAMN18650260 | DY06 | DY06 | DY_06_1.fq.gz | DY_06_2.fq.gz |
| SRR14179685 | SRP313870 | new | PRJNA716079 | SAMN18650261 | DY07 | DY07 | DY_07_1.fq.gz | DY_07_2.fq.gz |
| SRR14179684 | SRP313870 | new | PRJNA716079 | SAMN18650262 | DY08 | DY08 | DY_08_1.fq.gz | DY_08_2.fq.gz |
| SRR14179683 | SRP313870 | new | PRJNA716079 | SAMN18650263 | DY09 | DY09 | DY_09_1.fq.gz | DY_09_2.fq.gz |
| SRR14179682 | SRP313870 | new | PRJNA716079 | SAMN18650264 | DY10 | DY10 | DY_10_1.fq.gz | DY_10_2.fq.gz |
| SRR14179694 | SRP313870 | new | PRJNA716079 | SAMN18650265 | DY11 | DY11 | DY_11_1.fq.gz | DY_11_2.fq.gz |
| SRR14179693 | SRP313870 | new | PRJNA716079 | SAMN18650266 | DY12 | DY12 | DY_12_1.fq.gz | DY_12_2.fq.gz |
| SRR14179692 | SRP313870 | new | PRJNA716079 | SAMN18650267 | DY13 | DY13 | DY_13_1.fq.gz | DY_13_2.fq.gz |
| SRR14179691 | SRP313870 | new | PRJNA716079 | SAMN18650268 | DY14 | DY14 | DY_14_1.fq.gz | DY_14_2.fq.gz |
| SRR14179690 | SRP313870 | new | PRJNA716079 | SAMN18650269 | DY15 | DY15 | DY_15_1.fq.gz | DY_15_2.fq.gz |
| SRR14193926 | SRP313870 | new | PRJNA716079 | SAMN18677707 | PA01 | PA01 | PA_01_1.fq.gz | PA_01_2.fq.gz |
| SRR14193925 | SRP313870 | new | PRJNA716079 | SAMN18677708 | PA02 | PA02 | PA_02_1.fq.gz | PA_02_2.fq.gz |
| SRR14193919 | SRP313870 | new | PRJNA716079 | SAMN18677709 | PA03 | PA03 | PA_03_1.fq.gz | PA_03_2.fq.gz |
| SRR14193918 | SRP313870 | new | PRJNA716079 | SAMN18677710 | PA04 | PA04 | PA_04_1.fq.gz | PA_04_2.fq.gz |
| SRR14193917 | SRP313870 | new | PRJNA716079 | SAMN18677711 | PA05 | PA05 | PA_05_1.fq.gz | PA_05_2.fq.gz |
| SRR14193916 | SRP313870 | new | PRJNA716079 | SAMN18677712 | PA06 | PA06 | PA_06_1.fq.gz | PA_06_2.fq.gz |
| SRR14193915 | SRP313870 | new | PRJNA716079 | SAMN18677713 | PA07 | PA07 | PA_07_1.fq.gz | PA_07_2.fq.gz |
| SRR14193914 | SRP313870 | new | PRJNA716079 | SAMN18677714 | PA08 | PA08 | PA_08_1.fq.gz | PA_08_2.fq.gz |
| SRR14193913 | SRP313870 | new | PRJNA716079 | SAMN18677715 | PA09 | PA09 | PA_09_1.fq.gz | PA_09_2.fq.gz |
| SRR14193912 | SRP313870 | new | PRJNA716079 | SAMN18677716 | PA10 | PA10 | PA_10_1.fq.gz | PA_10_2.fq.gz |
| SRR14193924 | SRP313870 | new | PRJNA716079 | SAMN18677717 | PA11 | PA11 | PA_11_1.fq.gz | PA_11_2.fq.gz |
| SRR14193923 | SRP313870 | new | PRJNA716079 | SAMN18677718 | PA12 | PA12 | PA_12_1.fq.gz | PA_12_2.fq.gz |
| SRR14193922 | SRP313870 | new | PRJNA716079 | SAMN18677719 | PA13 | PA13 | PA_13_1.fq.gz | PA_13_2.fq.gz |
| SRR14193921 | SRP313870 | new | PRJNA716079 | SAMN18677720 | PA14 | PA14 | PA_14_1.fq.gz | PA_14_2.fq.gz |
| SRR14193920 | SRP313870 | new | PRJNA716079 | SAMN18677721 | PA15 | PA15 | PA_15_1.fq.gz | PA_15_2.fq.gz |
| SRR14202282 | SRP313870 | new | PRJNA716079 | SAMN18689749 | SD01 | SD01 | SD_01_1.fq.gz | SD_01_2.fq.gz |
| SRR14202281 | SRP313870 | new | PRJNA716079 | SAMN18689750 | SD02 | SD02 | SD_02_1.fq.gz | SD_02_2.fq.gz |
| SRR14202275 | SRP313870 | new | PRJNA716079 | SAMN18689751 | SD03 | SD03 | SD_03_1.fq.gz | SD_03_2.fq.gz |
| SRR14202274 | SRP313870 | new | PRJNA716079 | SAMN18689752 | SD04 | SD04 | SD_04_1.fq.gz | SD_04_2.fq.gz |
| SRR14202273 | SRP313870 | new | PRJNA716079 | SAMN18689753 | SD05 | SD05 | SD_05_1.fq.gz | SD_05_2.fq.gz |
| SRR14202272 | SRP313870 | new | PRJNA716079 | SAMN18689754 | SD06 | SD06 | SD_06_1.fq.gz | SD_06_2.fq.gz |
| SRR14202271 | SRP313870 | new | PRJNA716079 | SAMN18689755 | SD07 | SD07 | SD_07_1.fq.gz | SD_07_2.fq.gz |
| SRR14202270 | SRP313870 | new | PRJNA716079 | SAMN18689756 | SD08 | SD08 | SD_08_1.fq.gz | SD_08_2.fq.gz |
| SRR14202269 | SRP313870 | new | PRJNA716079 | SAMN18689757 | SD09 | SD09 | SD_09_1.fq.gz | SD_09_2.fq.gz |
| SRR14202268 | SRP313870 | new | PRJNA716079 | SAMN18689758 | SD10 | SD10 | SD_10_1.fq.gz | SD_10_2.fq.gz |
| SRR14202280 | SRP313870 | new | PRJNA716079 | SAMN18689759 | SD11 | SD11 | SD_11_1.fq.gz | SD_11_2.fq.gz |
| SRR14202279 | SRP313870 | new | PRJNA716079 | SAMN18689760 | SD12 | SD12 | SD_12_1.fq.gz | SD_12_2.fq.gz |
| SRR14202278 | SRP313870 | new | PRJNA716079 | SAMN18689761 | SD13 | SD13 | SD_13_1.fq.gz | SD_13_2.fq.gz |
| SRR14202277 | SRP313870 | new | PRJNA716079 | SAMN18689762 | SD14 | SD14 | SD_14_1.fq.gz | SD_14_2.fq.gz |
| SRR14202276 | SRP313870 | new | PRJNA716079 | SAMN18689763 | SD15 | SD15 | SD_15_1.fq.gz | SD_15_2.fq.gz |
| SRR14213607 | SRP313870 | new | PRJNA716079 | SAMN18715863 | SQ01 | SQ01 | SQ_01_1.fq.gz | SQ_01_2.fq.gz |
| SRR14213606 | SRP313870 | new | PRJNA716079 | SAMN18715864 | SQ02 | SQ02 | SQ_02_1.fq.gz | SQ_02_2.fq.gz |
| SRR14213600 | SRP313870 | new | PRJNA716079 | SAMN18715865 | SQ03 | SQ03 | SQ_03_1.fq.gz | SQ_03_2.fq.gz |
| SRR14213599 | SRP313870 | new | PRJNA716079 | SAMN18715866 | SQ04 | SQ04 | SQ_04_1.fq.gz | SQ_04_2.fq.gz |
| SRR14213598 | SRP313870 | new | PRJNA716079 | SAMN18715867 | SQ05 | SQ05 | SQ_05_1.fq.gz | SQ_05_2.fq.gz |
| SRR14213597 | SRP313870 | new | PRJNA716079 | SAMN18715868 | SQ06 | SQ06 | SQ_06_1.fq.gz | SQ_06_2.fq.gz |
| SRR14213596 | SRP313870 | new | PRJNA716079 | SAMN18715869 | SQ07 | SQ07 | SQ_07_1.fq.gz | SQ_07_2.fq.gz |
| SRR14213595 | SRP313870 | new | PRJNA716079 | SAMN18715870 | SQ08 | SQ08 | SQ_08_1.fq.gz | SQ_08_2.fq.gz |
| SRR14213594 | SRP313870 | new | PRJNA716079 | SAMN18715871 | SQ09 | SQ09 | SQ_09_1.fq.gz | SQ_09_2.fq.gz |
| SRR14213593 | SRP313870 | new | PRJNA716079 | SAMN18715872 | SQ10 | SQ10 | SQ_10_1.fq.gz | SQ_10_2.fq.gz |
| SRR14213605 | SRP313870 | new | PRJNA716079 | SAMN18715873 | SQ11 | SQ11 | SQ_11_1.fq.gz | SQ_11_2.fq.gz |
| SRR14213604 | SRP313870 | new | PRJNA716079 | SAMN18715874 | SQ12 | SQ12 | SQ_12_1.fq.gz | SQ_12_2.fq.gz |
| SRR14213603 | SRP313870 | new | PRJNA716079 | SAMN18715875 | SQ13 | SQ13 | SQ_13_1.fq.gz | SQ_13_2.fq.gz |
| SRR14213602 | SRP313870 | new | PRJNA716079 | SAMN18715876 | SQ14 | SQ14 | SQ_14_1.fq.gz | SQ_14_2.fq.gz |
| SRR14213601 | SRP313870 | new | PRJNA716079 | SAMN18715877 | SQ15 | SQ15 | SQ_15_1.fq.gz | SQ_15_2.fq.gz |
| SRR14308561 | SRP313870 | new | PRJNA716079 | SAMN18737686 | YH01 | YH01 | YH_01_1.fq.gz | YH_01_2.fq.gz |
| SRR14308560 | SRP313870 | new | PRJNA716079 | SAMN18737687 | YH02 | YH02 | YH_02_1.fq.gz | YH_02_2.fq.gz |
| SRR14308569 | SRP313870 | new | PRJNA716079 | SAMN18737688 | YH03 | YH03 | YH_03_1.fq.gz | YH_03_2.fq.gz |
| SRR14308568 | SRP313870 | new | PRJNA716079 | SAMN18737689 | YH04 | YH04 | YH_04_1.fq.gz | YH_04_2.fq.gz |
| SRR14308567 | SRP313870 | new | PRJNA716079 | SAMN18737690 | YH05 | YH05 | YH_05_1.fq.gz | YH_05_2.fq.gz |
| SRR14308566 | SRP313870 | new | PRJNA716079 | SAMN18737691 | YH06 | YH06 | YH_06_1.fq.gz | YH_06_2.fq.gz |
| SRR14308565 | SRP313870 | new | PRJNA716079 | SAMN18737692 | YH07 | YH07 | YH_07_1.fq.gz | YH_07_2.fq.gz |
| SRR14308564 | SRP313870 | new | PRJNA716079 | SAMN18737693 | YH08 | YH08 | YH_08_1.fq.gz | YH_08_2.fq.gz |
| SRR14308563 | SRP313870 | new | PRJNA716079 | SAMN18737694 | YH09 | YH09 | YH_09_1.fq.gz | YH_09_2.fq.gz |
| SRR14308562 | SRP313870 | new | PRJNA716079 | SAMN18737695 | YH10 | YH10 | YH_10_1.fq.gz | YH_10_2.fq.gz |
| SRR14308559 | SRP313870 | new | PRJNA716079 | SAMN18737696 | YH11 | YH11 | YH_11_1.fq.gz | YH_11_2.fq.gz |
| SRR14308558 | SRP313870 | new | PRJNA716079 | SAMN18737698 | YH13 | YH13 | YH_13_1.fq.gz | YH_13_2.fq.gz |
| SRR14308571 | SRP313870 | new | PRJNA716079 | SAMN18737699 | YH14 | YH14 | YH_14_1.fq.gz | YH_14_2.fq.gz |
| SRR14308570 | SRP313870 | new | PRJNA716079 | SAMN18737700 | YH15 | YH15 | YH_15_1.fq.gz | YH_15_2.fq.gz |
| SRR14308557 | SRP313870 | new | PRJNA716079 | SAMN18720204 | XS01 | XS01 | XS_01_1.fq.gz | XS_01_2.fq.gz |
| SRR14308556 | SRP313870 | new | PRJNA716079 | SAMN18720205 | XS02 | XS02 | XS_02_1.fq.gz | XS_02_2.fq.gz |
| SRR14308550 | SRP313870 | new | PRJNA716079 | SAMN18720206 | XS03 | XS03 | XS_03_1.fq.gz | XS_03_2.fq.gz |
| SRR14308549 | SRP313870 | new | PRJNA716079 | SAMN18720207 | XS04 | XS04 | XS_04_1.fq.gz | XS_04_2.fq.gz |
| SRR14308548 | SRP313870 | new | PRJNA716079 | SAMN18720208 | XS05 | XS05 | XS_05_1.fq.gz | XS_05_2.fq.gz |
| SRR14308547 | SRP313870 | new | PRJNA716079 | SAMN18720210 | XS07 | XS07 | XS_07_1.fq.gz | XS_07_2.fq.gz |
| SRR14308546 | SRP313870 | new | PRJNA716079 | SAMN18720211 | XS08 | XS08 | XS_08_1.fq.gz | XS_08_2.fq.gz |
| SRR14308545 | SRP313870 | new | PRJNA716079 | SAMN18720212 | XS09 | XS09 | XS_09_1.fq.gz | XS_09_2.fq.gz |
| SRR14308544 | SRP313870 | new | PRJNA716079 | SAMN18720213 | XS10 | XS10 | XS_10_1.fq.gz | XS_10_2.fq.gz |
| SRR14308555 | SRP313870 | new | PRJNA716079 | SAMN18720214 | XS11 | XS11 | XS_11_1.fq.gz | XS_11_2.fq.gz |
| SRR14308554 | SRP313870 | new | PRJNA716079 | SAMN18720215 | XS12 | XS12 | XS_12_1.fq.gz | XS_12_2.fq.gz |
| SRR14308553 | SRP313870 | new | PRJNA716079 | SAMN18720216 | XS13 | XS13 | XS_13_1.fq.gz | XS_13_2.fq.gz |
| SRR14308552 | SRP313870 | new | PRJNA716079 | SAMN18720217 | XS14 | XS14 | XS_14_1.fq.gz | XS_14_2.fq.gz |
| SRR14308551 | SRP313870 | new | PRJNA716079 | SAMN18720218 | XS15 | XS15 | XS_15_1.fq.gz | XS_15_2.fq.gz |
| SRR14309711 | SRP313870 | new | PRJNA716079 | SAMN18650533 | HK01 | HK01 | HK_01_1.fq.gz | HK_01_2.fq.gz |
| SRR14309719 | SRP313870 | new | PRJNA716079 | SAMN18650535 | HK03 | HK03 | HK_03_1.fq.gz | HK_03_2.fq.gz |
| SRR14309718 | SRP313870 | new | PRJNA716079 | SAMN18650536 | HK04 | HK04 | HK_04_1.fq.gz | HK_04_2.fq.gz |
| SRR14309717 | SRP313870 | new | PRJNA716079 | SAMN18650537 | HK05 | HK05 | HK_05_1.fq.gz | HK_05_2.fq.gz |
| SRR14309716 | SRP313870 | new | PRJNA716079 | SAMN18650538 | HK06 | HK06 | HK_06_1.fq.gz | HK_06_2.fq.gz |
| SRR14309715 | SRP313870 | new | PRJNA716079 | SAMN18650539 | HK07 | HK07 | HK_07_1.fq.gz | HK_07_2.fq.gz |
| SRR14309714 | SRP313870 | new | PRJNA716079 | SAMN18650540 | HK08 | HK08 | HK_08_1.fq.gz | HK_08_2.fq.gz |
| SRR14309713 | SRP313870 | new | PRJNA716079 | SAMN18650541 | HK09 | HK09 | HK_09_1.fq.gz | HK_09_2.fq.gz |
| SRR14309712 | SRP313870 | new | PRJNA716079 | SAMN18650542 | HK10 | HK10 | HK_10_1.fq.gz | HK_10_2.fq.gz |
| SRR14309709 | SRP313870 | new | PRJNA716079 | SAMN18650543 | HK11 | HK11 | HK_11_1.fq.gz | HK_11_2.fq.gz |
| SRR14309708 | SRP313870 | new | PRJNA716079 | SAMN18650544 | HK12 | HK12 | HK_12_1.fq.gz | HK_12_2.fq.gz |
| SRR14309722 | SRP313870 | new | PRJNA716079 | SAMN18650545 | HK13 | HK13 | HK_13_1.fq.gz | HK_13_2.fq.gz |
| SRR14309721 | SRP313870 | new | PRJNA716079 | SAMN18650546 | HK14 | HK14 | HK_14_1.fq.gz | HK_14_2.fq.gz |
| SRR14309720 | SRP313870 | new | PRJNA716079 | SAMN18650547 | HK15 | HK15 | HK_15_1.fq.gz | HK_15_2.fq.gz |
